# Supplementary material for: Nanowire-based smart windows combining electro- and thermochromics for dynamic regulation of solar radiation
Source: Nat Commun. 2023 Jun 3;14:3231. doi: 10.1038/s41467-023-38353-4 (PMC10239468; doi:10.1038/s41467-023-38353-4)
Supplement: Supplementary file 1 — Supplementary Information [file 41467_2023_38353_MOESM1_ESM.docx]

Supplementary Information

**Nanowire-based smart windows combining electro- and thermochromics for dynamic regulation of solar radiation**

Si-Zhe Sheng^†1^, Jin-Long Wang^†2^, Bin Zhao^†3^, Zhen He^2^, Xue-Fei Feng^1^, Qi-Guo Shang^1^, Cheng Chen^1^, Gang Pei^3^, Jun Zhou^4^, Jian-Wei Liu*^1^ and Shu-Hong Yu*^1,2^

*^1^Department of Chemistry, Institute of Biomimetic Materials & Chemistry, Anhui Engineering Laboratory of Biomimetic Materials, Division of Nanomaterials & Chemistry, Hefei National Research Center for Physical Sciences at the Microscale, University of Science and Technology of China, Hefei 230026, China.*

*^2^Institute of Innovative Materials (I2M), Department of Materials Science and Engineering, Southern University of Science and Technology, Shenzhen 518055, China*

*^3^Department of Thermal Science and Energy Engineering, University of Science and Technology of China, Hefei, Anhui 230026, China*

*^4^Hefei National Research Center for Physical Sciences at the Microscale, University of Science and Technology of China, Hefei, Anhui 230026, China*

^†^These authors contributed equally to this work.

^*^Correspondence and requests for materials should be addressed to the author: Jian-Wei Liu ([jwliu13@ustc.edu.cn](mailto:jwliu13@ustc.edu.cn)) or Shu-Hong Yu ([shyu@ustc.edu.cn](mailto:shyu@ustc.edu.cn)).

**Supplementary note 1. The synthesis of Au NRs with** **multiple aspect ratios**

**Supplementary Table 1****. The synthesis conditions for Au NRs.**

|  | **CTAB** | **NaOL** | **AgNO_3_** | **HCl** | **Ascorbic Acid** | **Seed Solution** |
| --- | --- | --- | --- | --- | --- | --- |
| **Au NR-1** | **7.0 g** | **1.234 g** | **24 mL** | **2.1 mL** | **1.25 mL** | **0.8 mL** |
| **Au NR-2** | **7.0 g** | **1.234 g** | **24 mL** | **3.0 mL** | **1.25 mL** | **0.4 mL** |
| **Au NR-3** | **7.0 g** | **1.234 g** | **24 mL** | **3.6 mL** | **1.25 mL** | **0.4 mL** |
| **Au NR-4** | **7.0 g** | **1.234 g** | **24 mL** | **5.0 mL** | **1.25 mL** | **0.8 mL** |
| **Au NR-5** | **7.0 g** | **1.234 g** | **24 mL** | **6.4 mL** | **1.25 mL** | **0.8 mL** |


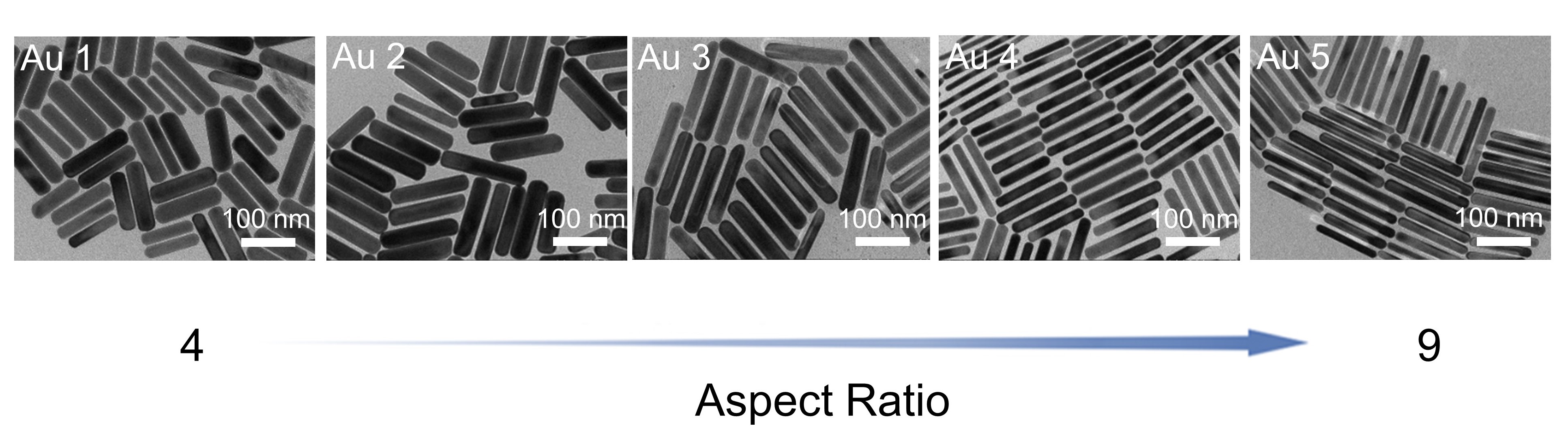


**Supplementary Figure 1.** **TEM images of Au NRs.** The aspect ratios of Au NRs ranges from 4 to 9.


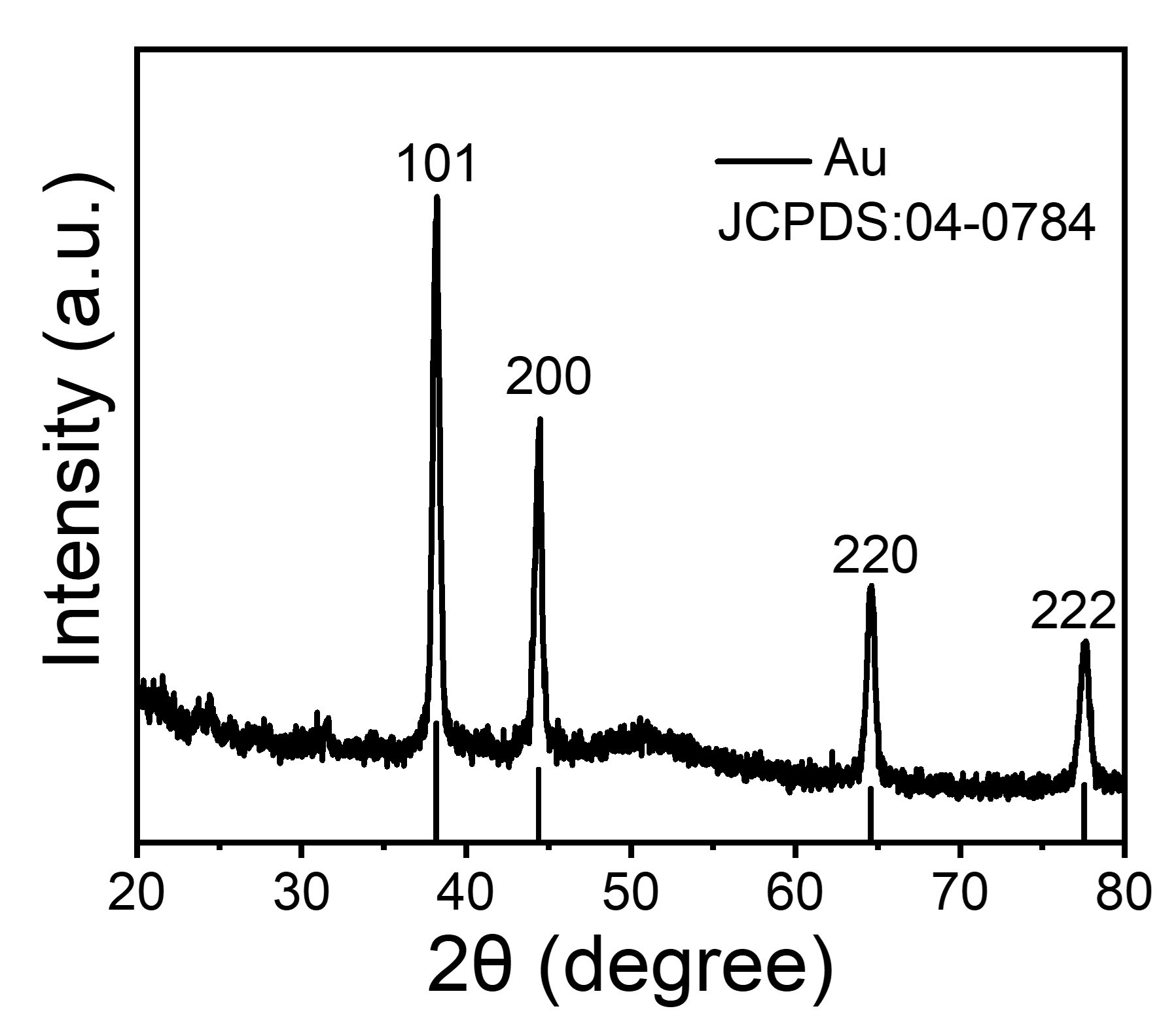


**Supplementary Figure 2. XRD patterns of Au NRs.** Source data are provided as a Source Data file.

**Supplementary note 2. The synthesis of Ag NWs and W_18_O_49_ NWs**


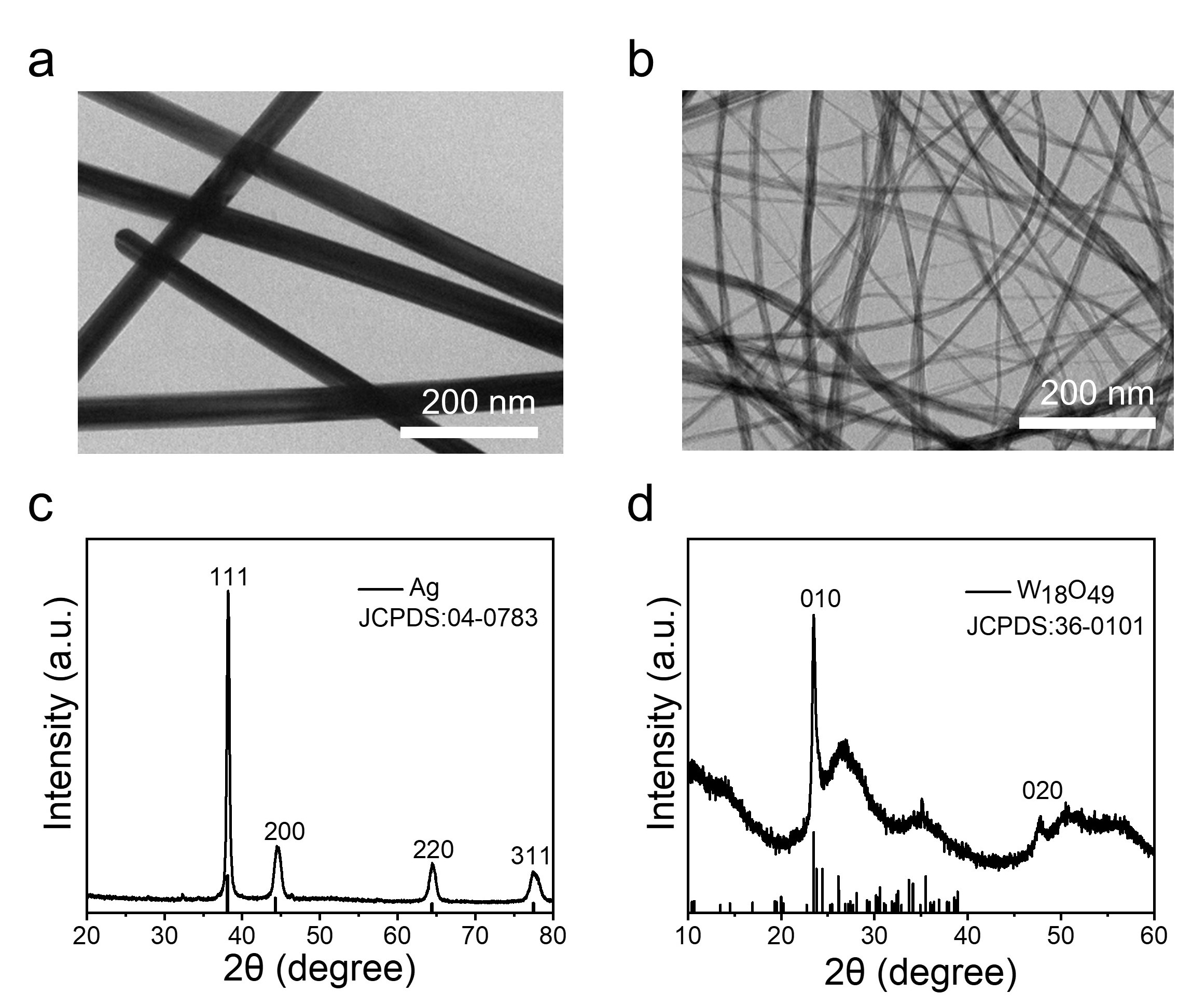


**Supplementary Figure 3. The synthesis of nanowires. a, b,** TEM images of Ag and W_18_O_49_ NWs. **c, d,** XRD patterns of Ag and W_18_O_49_ NWs. Source data are provided as a Source Data file.

**Supplementary note 3. The Ordered arrangement network structure of one-dimension materials**


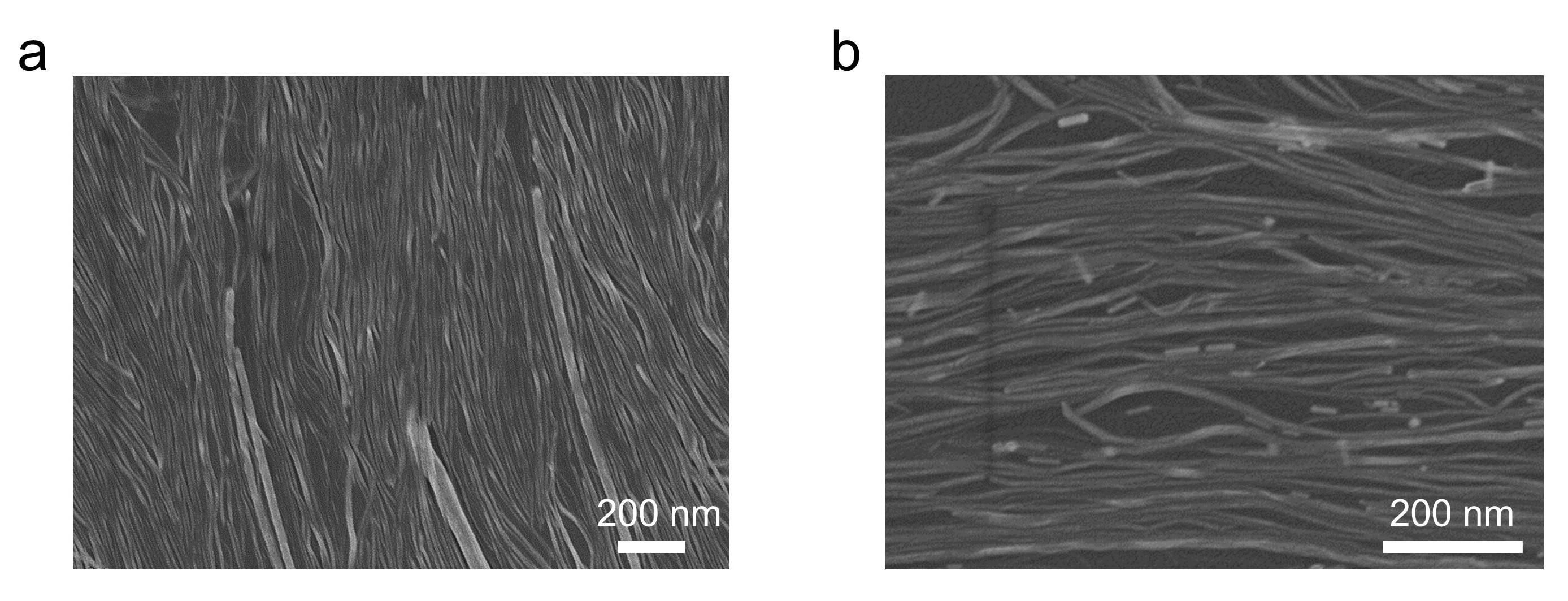


**Supplementary Figure 4. Ordered arrangement of monolayer nanowire assemblies. a,** SEM image of W_18_O_49_/Ag monolayer layer. **b,** SEM image of W_18_O_49_/Au monolayer layer.


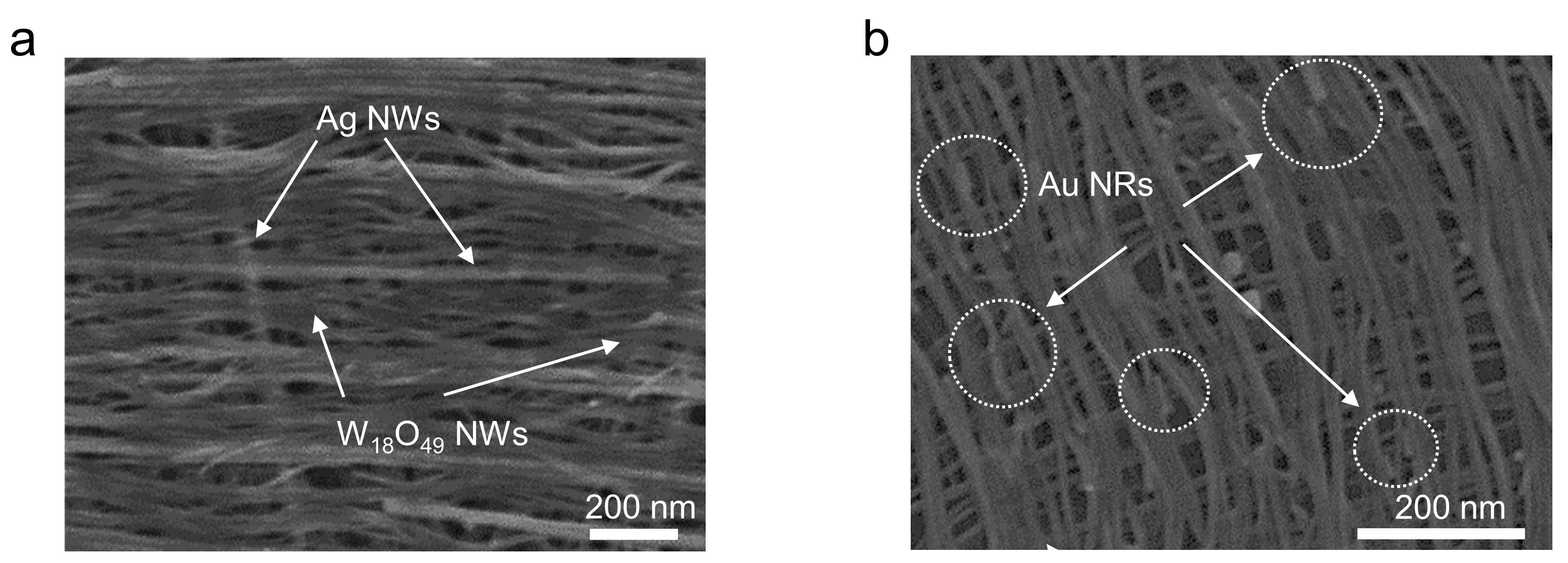


**Supplementary Figure 5. The network structure of nanowire assemblies. a,** SEM image of W_18_O_49_/Ag network structure. **b,** SEM image of W_18_O_49_/Au network structure.

.

**Supplementary note 4.** **Comparison of ordered and disordered structures in SLE films**


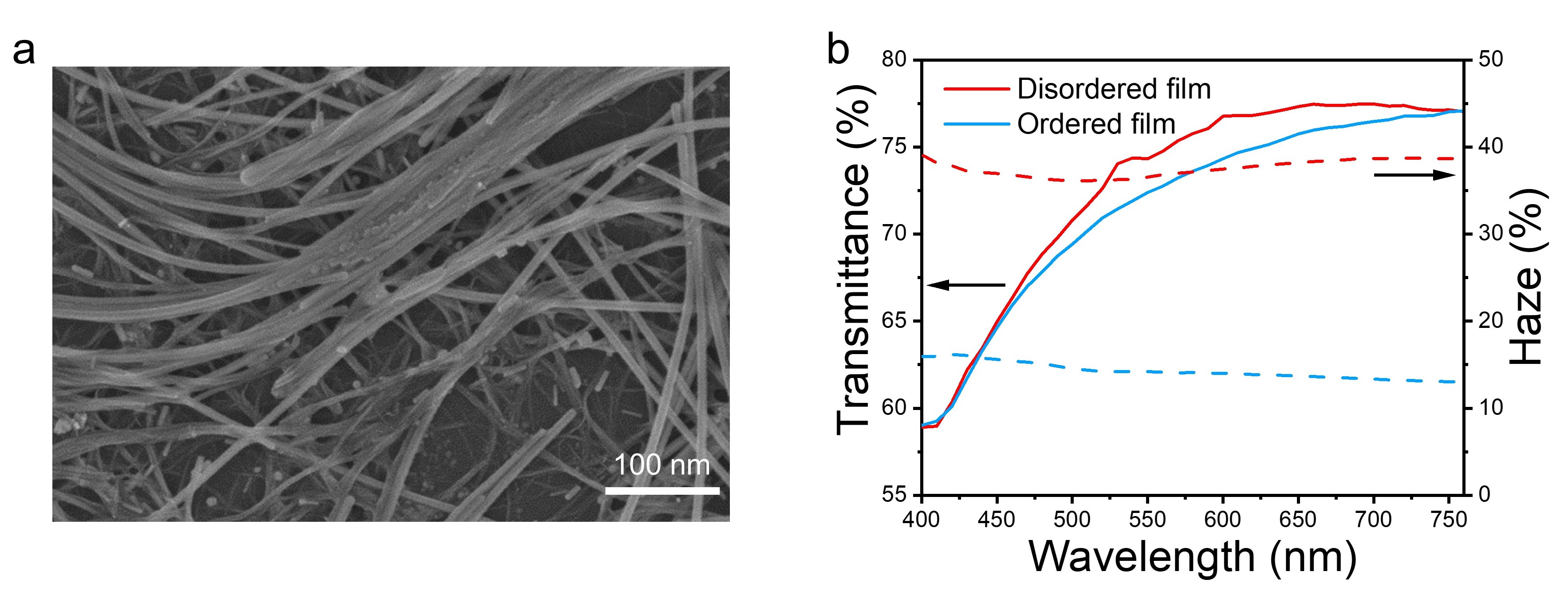


**Supplementary Figure 6.** **Comparison of ordered and disordered structures. a,** SEM image of disorder spraying W_18_O_49_/Ag/Au film. **b,** Optical transmittance and haze of the ordered and disordered structures in SLE films. Source data are provided as a Source Data file.

**Supplementary note 5.** **Preparation and characterization of EC films with different layers**


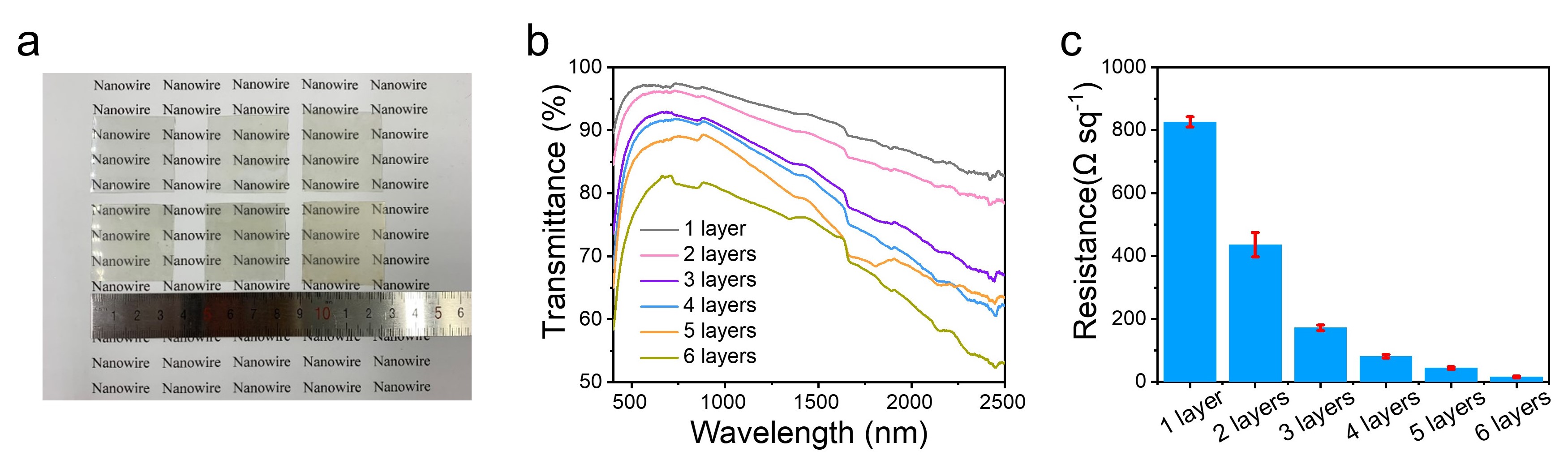


**Supplementary Figure 7. The EC films with different layers. a,** Photograph of the as-prepared conductive electrochromic W_18_O_49_/Ag NWs films with different layers of 1, 2, 3, 4, 5, and 6 (from left to right, from top to bottom). **b, c,** Optical transmittance spectra and corresponding sheet resistance of the as-prepared electrochromic W_18_O_49_/Ag NWs films. The corresponding error bar represents the standard deviation. For calculation, the cooling effect of each sample was measured five times. Source data are provided as a Source Data file.

**Supplementary Table 2.** Integrated optical transmittance and solar Irradiance transmittance of the as-prepared electrochromic W_18_O_49_/Ag NWs films with different layers of 1, 2, 3, 4, 5, and 6 in the VIS (400-760 nm), NIR-1 (760-1360 nm), NIR-2 (1360-2500 nm) and Sol (400-2500 nm) regions.

|  | ***T_VIS_*** | ***T_NIR-1_*** | ***T_NIR-2_*** | ***T_Sol_*** |
| --- | --- | --- | --- | --- |
| **1 layer** | 96.2% | 95.1% | 87.2% | 91.1% |
| **2 layers** | 94.4% | 93.2% | 83.7% | 88.3% |
| **3 layers** | 89.6% | 89.1% | 74.8% | 81.5% |
| **4 layers** | 88.7% | 88.2% | 71.2% | 80.6% |
| **5 layers** | 84.8% | 85.6% | 64.6% | 74.1% |
| **6 layers** | 77.6% | 79.3% | 68.7% | 73.3% |

**Supplementary note 6. Preparation of electrochromic and selective light absorption electrochromic films**


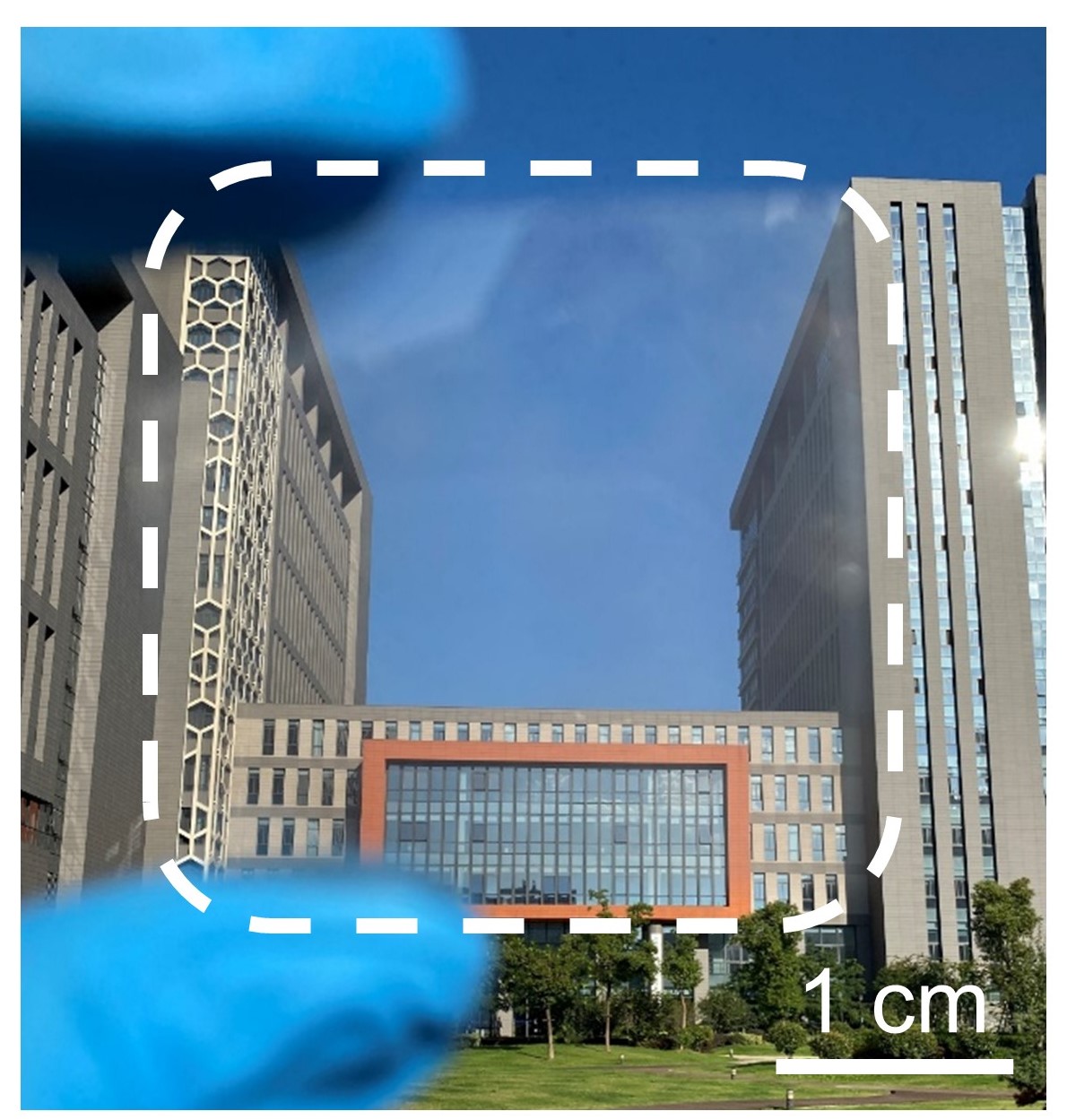


**Supplementary Figure 8.** **Photograph of electrochromic film.** The area covered with electrochromic (EC) film is denoted with the white imaginary line.

**Supplementary Table 3.** The preparation conditions of electrochromic and selective light absorption-electrochromic films.

|  | **EC** | **SLE1** | **SLE2** | **SLE3** | **SLE4** |
| --- | --- | --- | --- | --- | --- |
| **Au NR-1** | 0 mL | 0 mL | 0 mL | 0.33 mL | 0.20 mL |
| **Au NR-2** | 0 mL | 0 mL | 0.50 mL | 0 mL | 0.20 mL |
| **Au NR-3** | 0 mL | 1.00 mL | 0 mL | 0.33 mL | 0.20 mL |
| **Au NR-4** | 0 mL | 0 mL | 0.50 mL | 0 mL | 0.20 mL |
| **Au NR-5** | 0 mL | 0 mL | 0 mL | 0.33 mL | 0.20 mL |
| **Ag NW** | 0.60 mL | 0.60 mL | 0.60 mL | 0.60 mL | 0.60 mL |
| **W_18_O_49_ NW** | 1.50 mL | 1.50 mL | 1.50 mL | 1.50 mL | 1.50 mL |


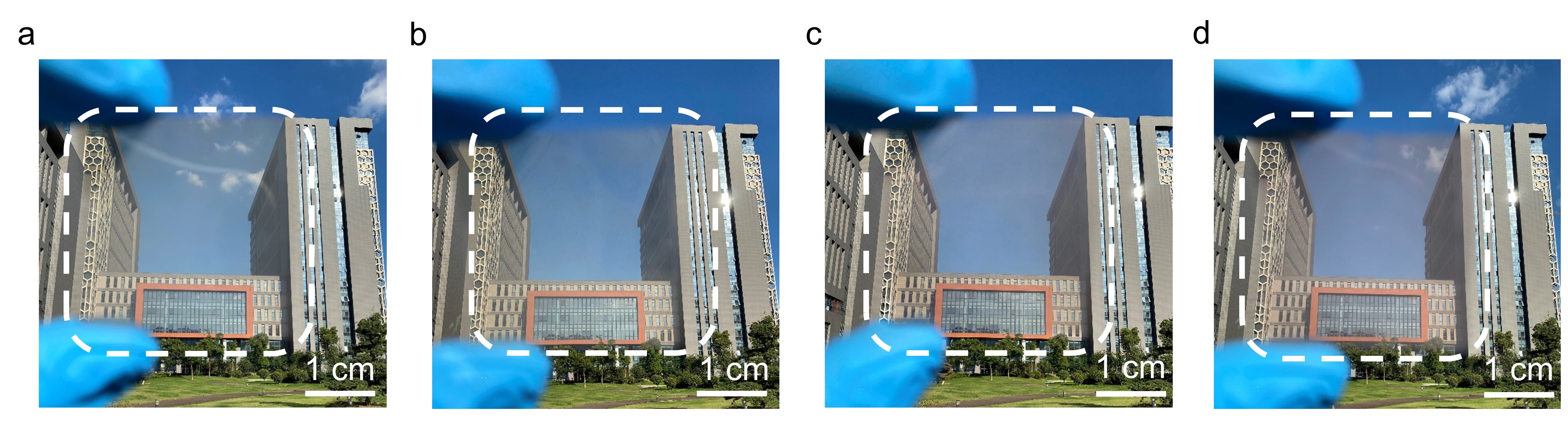


**Supplementary Figure 9. Photographs of selective light absorption-electrochromic films. a, b, c, d,** Selective light absorption-electrochromic films SLE1, SLE2, SLE3, and SLE4 films, the areas covered with SLE films are denoted with the white imaginary line.

**Supplementary note 7.** **Integrated optical and solar irradiance transmittance of EC and SLE films**

**Supplementary Table 4.** Integrated optical transmittance and solar Irradiance transmittance of EC, SLE1, SLE2, SLE3, and SLE4 films in the VIS, NIR-1, NIR-2, Sol regions.

|  | ***T_VIS_*** | ***T_NIR-1_*** | ***T_NIR-2_*** | ***T_Sol_*** | ***T_VIS_’*** | ***T_NIR-1_’*** | | ***T_NIR-2_’*** | ***T_Sol_’*** |
| --- | --- | --- | --- | --- | --- | --- | --- | --- | --- |
| **EC** | 90.2% | 86.7% | 78.4% | 80.5% | 90.3% | | 87.5% | 76.9% | 87.7% |
| **SLE1** | 71.3% | 58.6% | 65.3% | 66.4% | 71.6% | | 59.3% | 69.5% | 67.2% |
| **SLE2** | 70.3% | 61.7% | 60.4% | 62.2% | 70.5% | | 61.7% | 62.3% | 66.5% |
| **SLE3** | 71.8% | 55.4% | 57.7% | 60.1% | 71.9% | | 55.6% | 58.7% | 65.5% |
| **SLE4** | 71.0% | 54.6% | 56.8% | 59.3% | 71.1% | | 54.8% | 58.4% | 63.5% |

**Supplementary note 8. Preparation and characterization of SLE films with different layers**


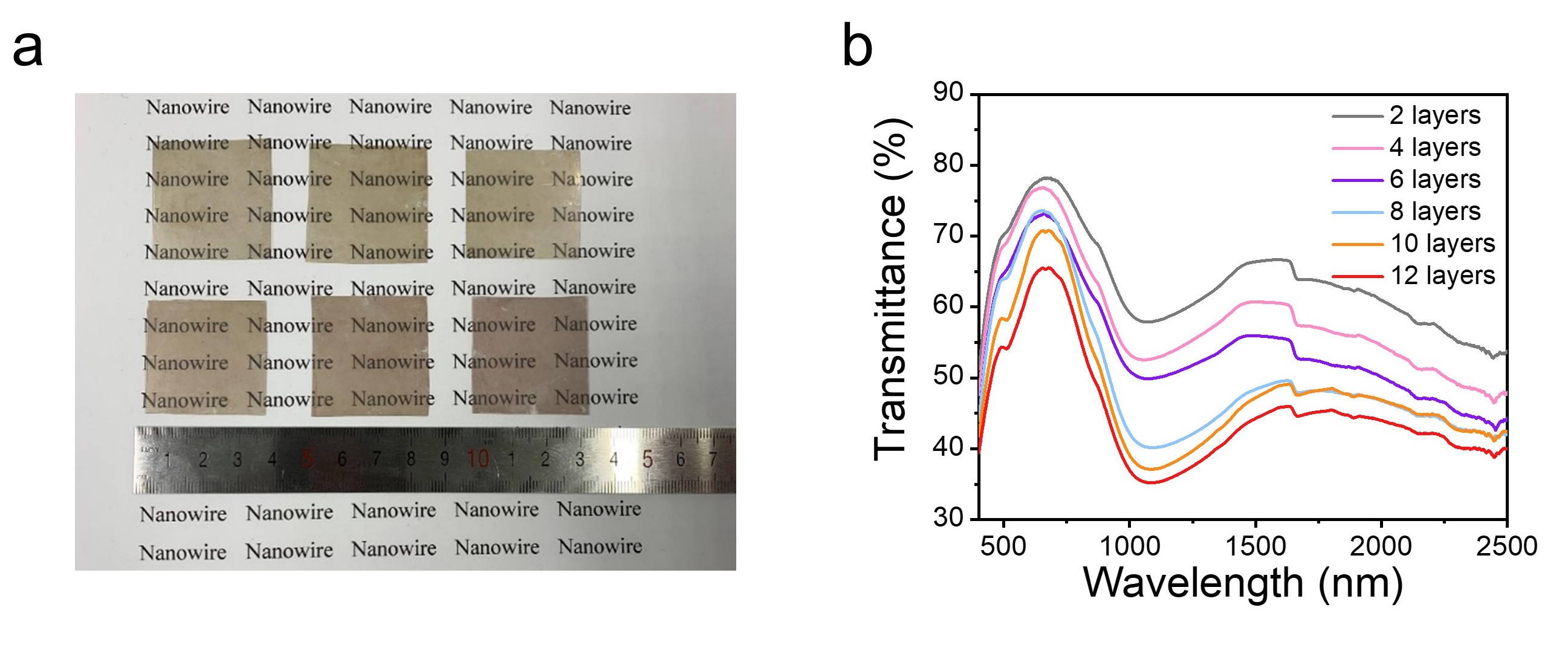


**Supplementary Figure 10. The SLE films with different layers. a,** Photograph of the as-prepared selective light absorption W_18_O_49_/Au NWs films with different layers of 2, 4, 6, 8, 10, and 12 (from left to right, from top to bottom). **b,** Optical transmittance spectra of the as-prepared selective light absorption W_18_O_49_/Au NWs films. Source data are provided as a Source Data file.

**Supplementary Table 5.** Integrated optical transmittance (*T*) and solar Irradiance transmittance (*T’*) of the as-prepared light absorption W_18_O_49_/Au NWs films with different layers of 2, 4, 6, 8, 10, and 12 in the VIS, NIR-1, NIR-2, Sol regions.

|  | ***T_VIS_*** | ***T_NIR-1_*** | ***T_NIR-2_*** | ***T_Sol_*** |
| --- | --- | --- | --- | --- |
| **2 layers** | 72.3% | 62.7% | 60.8% | 63.4% |
| **4 layers** | 71.8% | 57.5% | 54.6% | 58.2% |
| **6 layers** | 71.0% | 54.8% | 50.5% | 54.2% |
| **8 layers** | 70.3% | 48.1% | 47.5% | 51.3% |
| **10 layers** | 65.6% | 43.8% | 45.8% | 48.6% |
| **12 layers** | 59.8% | 41.2% | 43.0% | 45.2% |

**Supplementary note 9. The temperature of the indoor blackbody in the model chamber with a window installed with bare PC, EC, and SLE films under direct simulated sunlight**


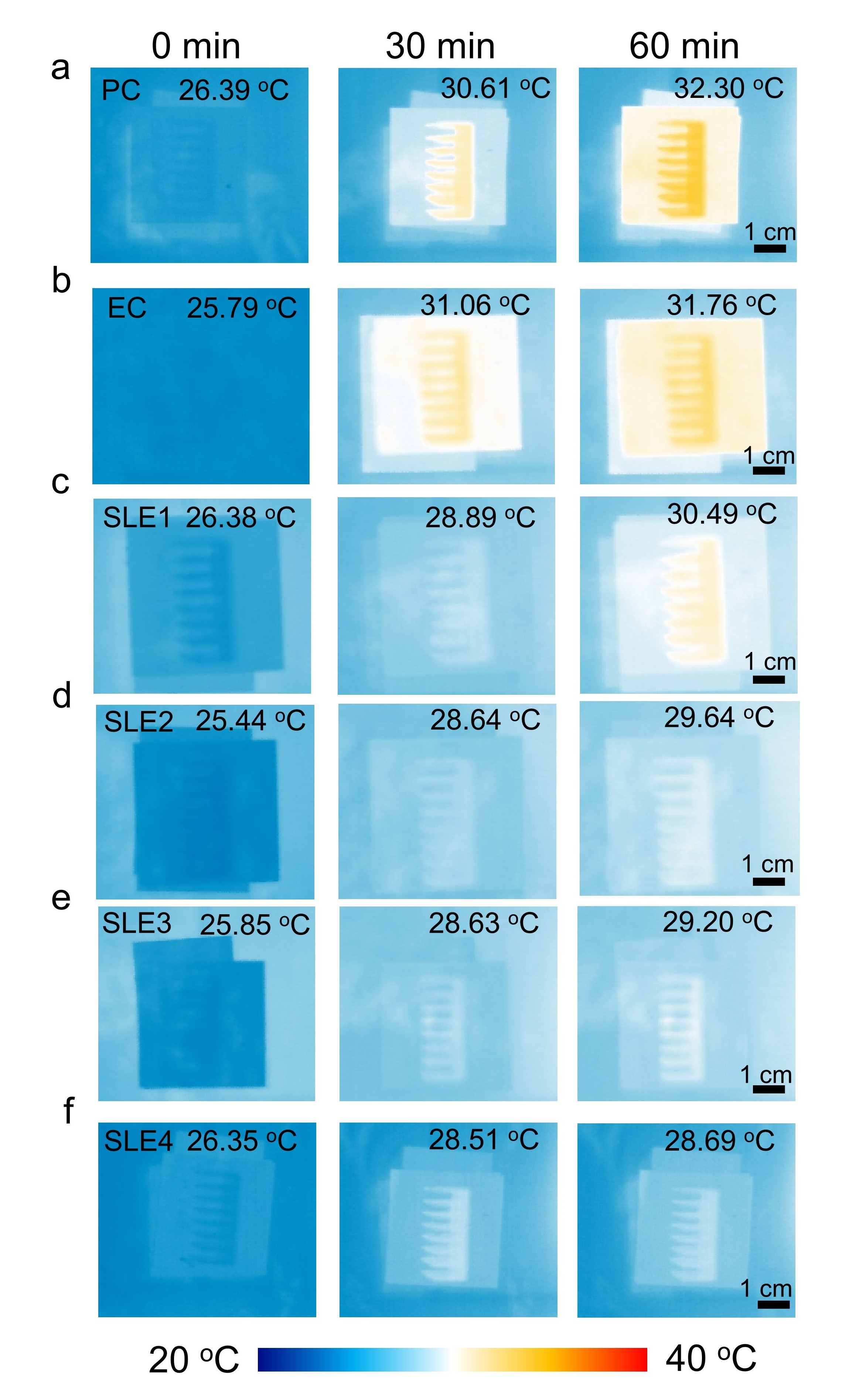


**Supplementary Figure 11. The cooling effect of different electrochromic windows. a-f,** The time-dependent infrared images of the indoor blackbody in the model chamber with a window installed with blank PC, EC, SLE1, SLE2, SLE3, and SLE4 films, respectively.

**Supplementary note 10. Electrochromic function of SLE4 films**


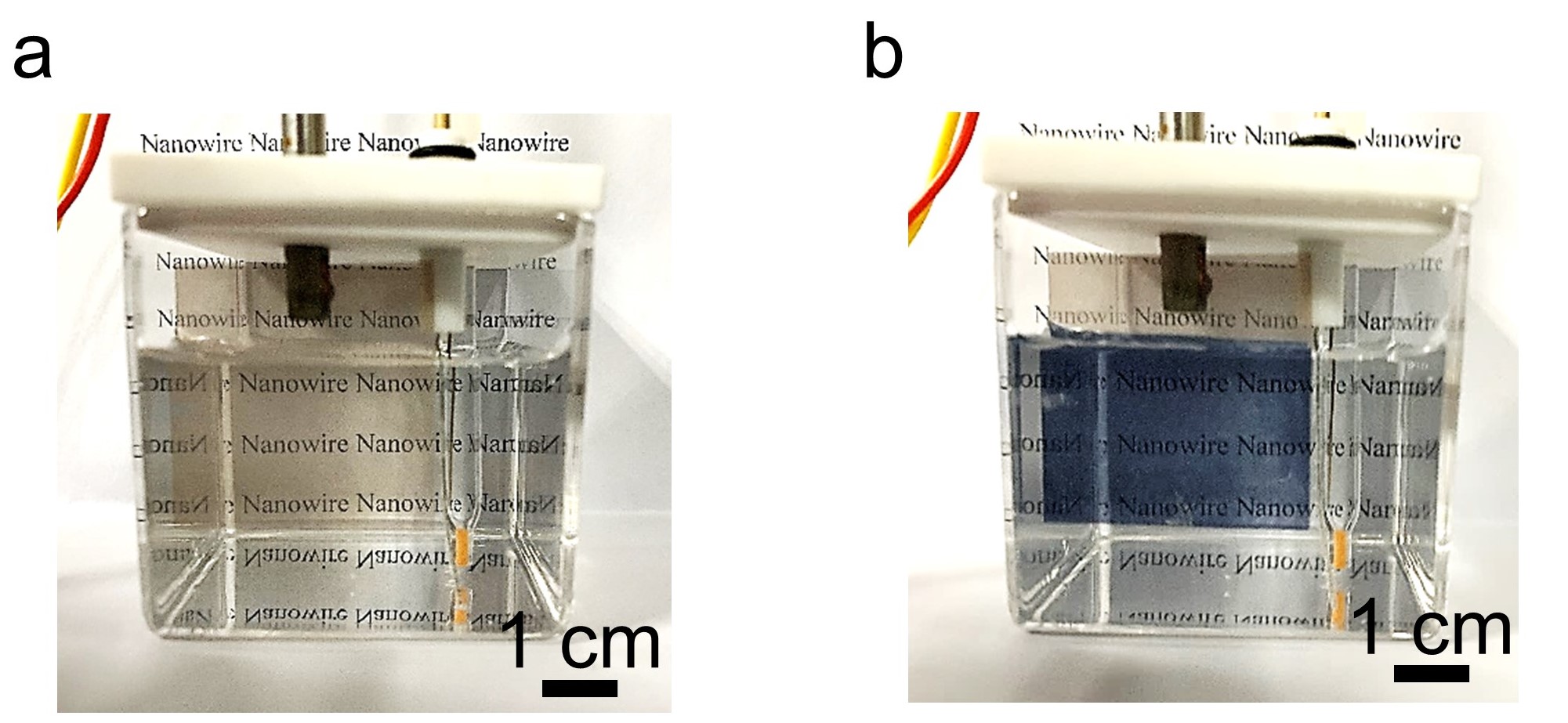


**Supplementary Figure 12. The color transformation of SLE4 films. a, b,** Photographs of the SLE4 film at bleached and colored states.

**Supplementary Table 6.** Integrated optical transmittance and solar Irradiance transmittance of EC and SLE4 films in the VIS and NIR-1 regions at bleached and colored states.

|  | ***T_VIS_*** | ***T_NIR-1_*** | ***T_VIS_’*** | ***T_NIR-1_’*** |
| --- | --- | --- | --- | --- |
| **EC bleached** | 90.2% | 86.7% | 90.3% | 87.5% |
| **EC colored** | 46.3% | 30.5% | 46.6% | 32.0% |
| **SLE4 bleached** | 70.3% | 48.1% | 70.5% | 49.0% |
| **SLE4 colored** | 29.6% | 9.1% | 29.8% | 9.4% |


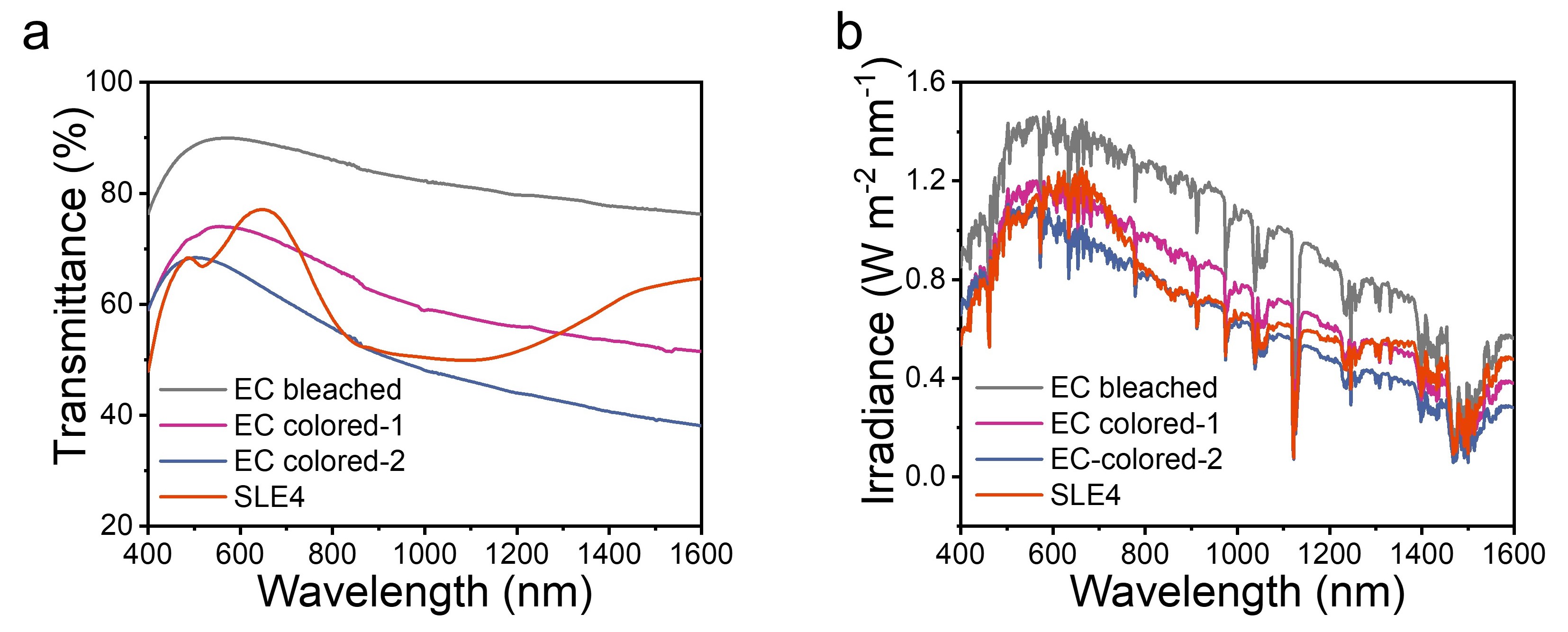


**Supplementary Figure 13.** **Comparison of selective spectral regulation of SLE4 and EC films. a,** The transmittance spectra of SLE4 and EC at different colored states. **b,** The transmitted solar irradiance of SLE4 and EC at different colored states. Source data are provided as a Source Data file.

**Supplementary Table 7.** Integrated optical transmittance and solar Irradiance transmittance of EC at bleached and colored states, compared to the SLE4 films in the VIS and NIR-1 regions.

|  | ***T_VIS_*** | ***T_NIR-1_*** | ***T_VIS_’*** | ***T_NIR-1_’*** |
| --- | --- | --- | --- | --- |
| **EC bleached** | 88.3% | 81.8% | 87.8% | 82.2% |
| **EC colored-1** | 71.0% | 59.3% | 70.9% | 59.7% |
| **EC colored-2** | 64.5% | 47.9% | 64.2% | 48.4% |
| **SLE4** | 70.3% | 48.1% | 70.5% | 49.0% |


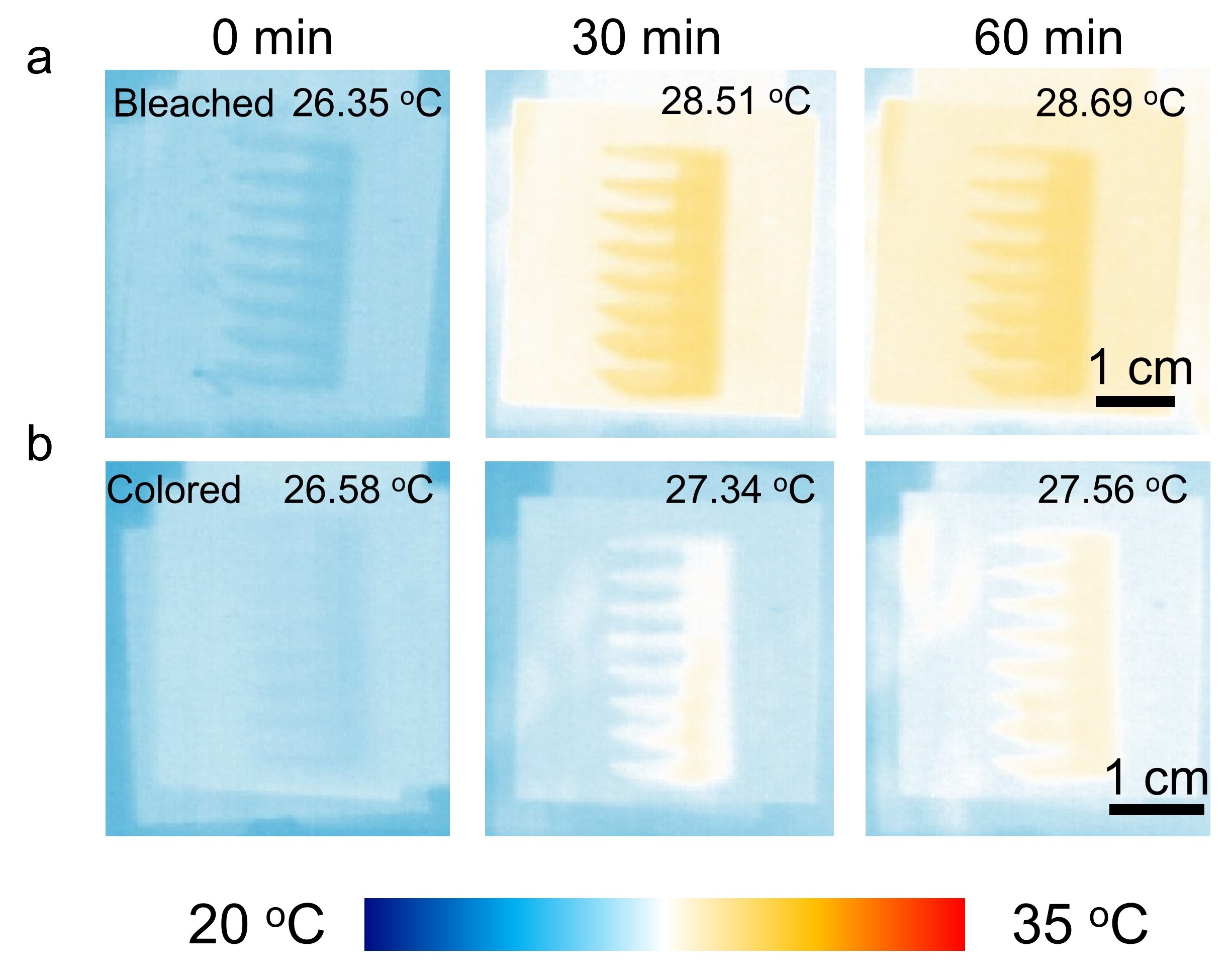


**Supplementary Figure 14. The cooling effect of SLE4 film at different states. a, b,** The time-dependent infrared images for the indoor blackbody in the model chamber with window installed with bleached and colored state of selective light absorption-electrochromic smart window solid devices.


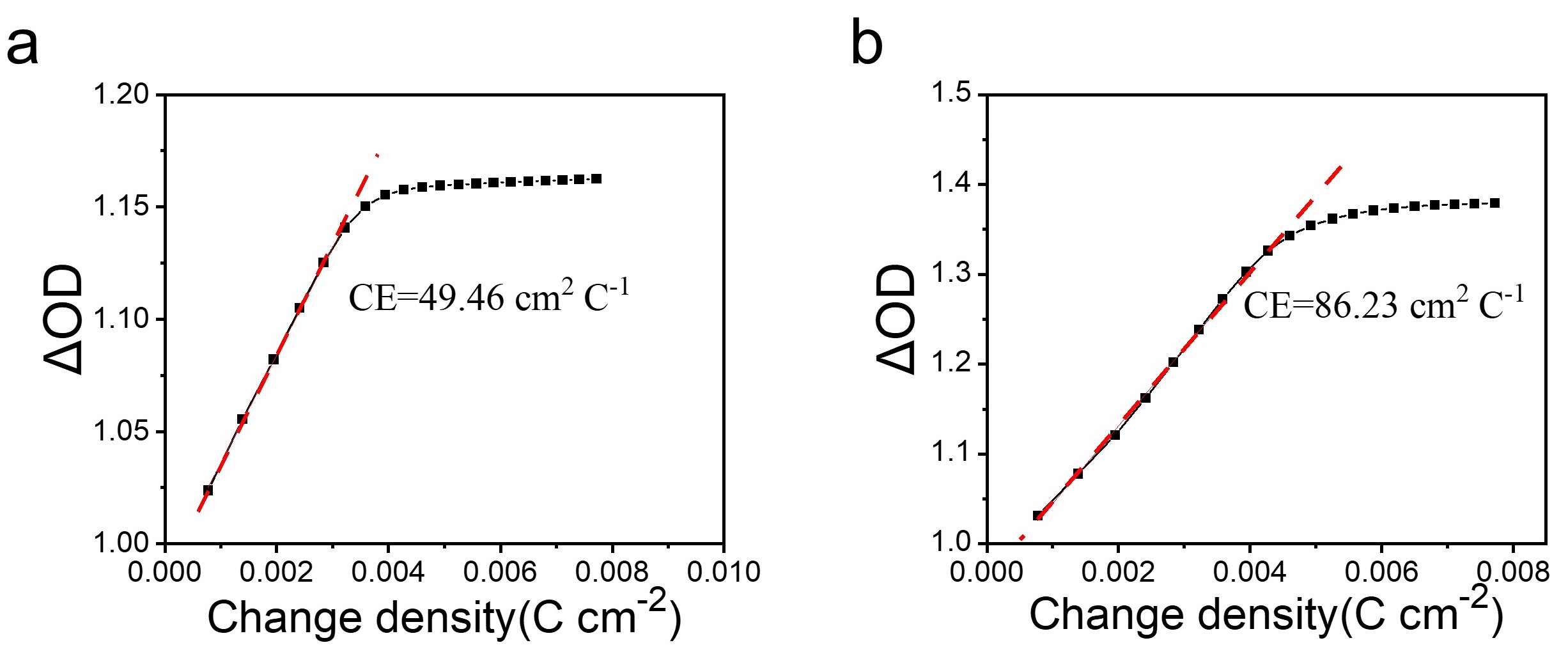


**Supplementary Figure 15. Coloration efficiency of the SLE4 film. a**, At 613.5nm. **b**, At 900 nm. Source data are provided as a Source Data file.

**Supplementary note 11. Electrochromic switching of SLE4 film**


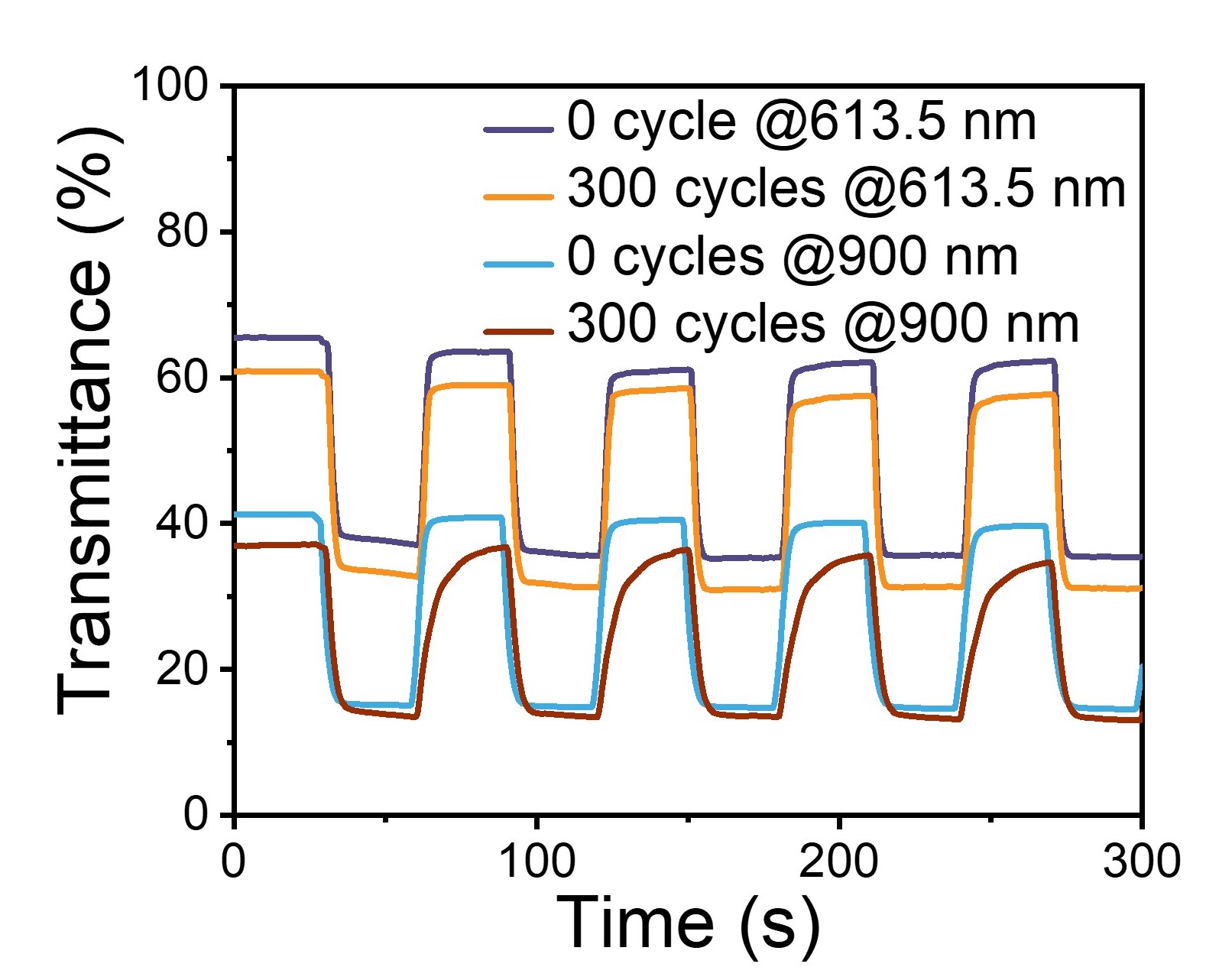


**Supplementary Figure 16. Electrochromic cyclic stability of SLE4 film.** The electrochromic switching for 300 cycles. Source data are provided as a Source Data file.

**Supplementary note 12. Stability of the cooling performance of SLE4 film**


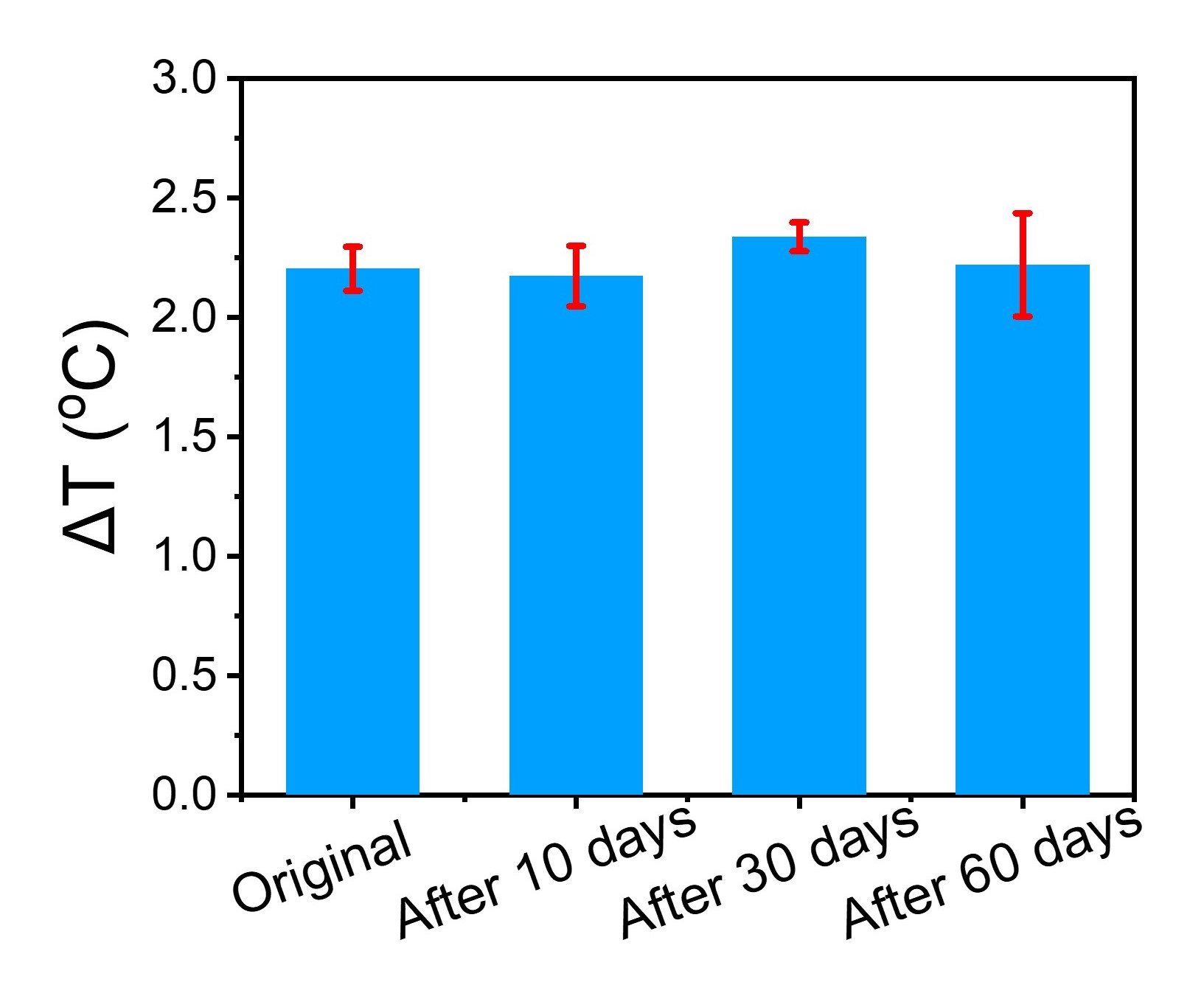


**Supplementary Figure 17.** **The stability of the cooling performance of SLE4 film.** The variation of cooling properties of SLE4 film exposed to air for the different time. The corresponding error bar represents the standard deviation. For calculation, the cooling effect of each sample was measured three times. Source data are provided as a Source Data file.

**Supplementary note 13. The synthesis of W doped VO_2_ NWs**


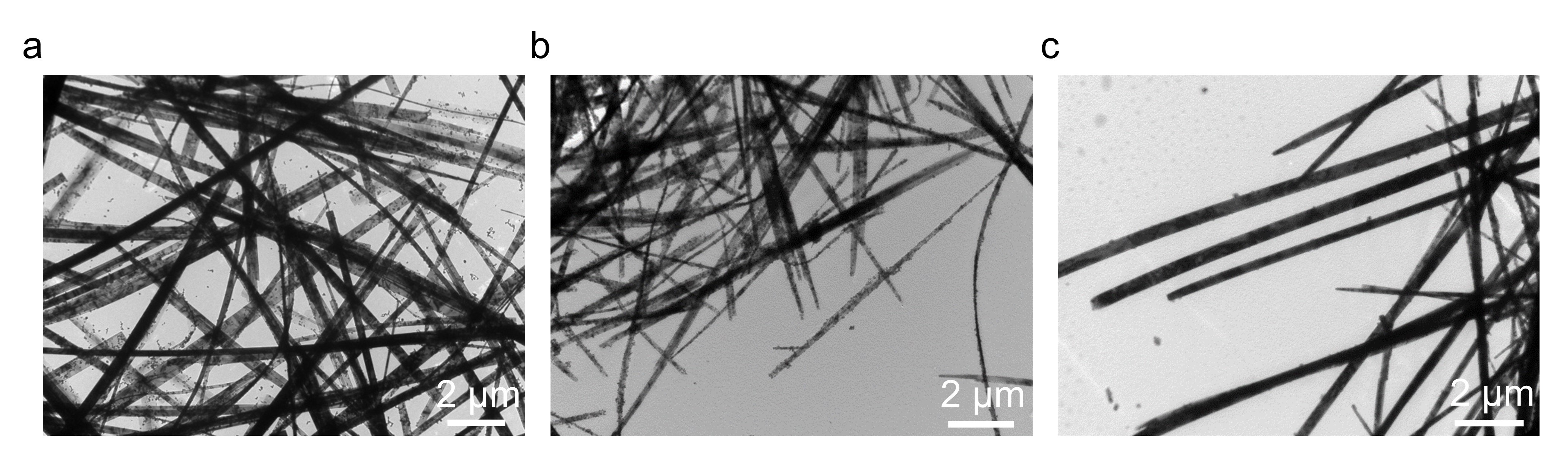


**Supplementary Figure 18. TEM images of different phase transition temperatures of W-doped VO_2_ NWs. a,** W-VO_2_-1 NWs. **b,** W-VO_2_-2 NWs. **c,** W-VO_2_-3 NWs.


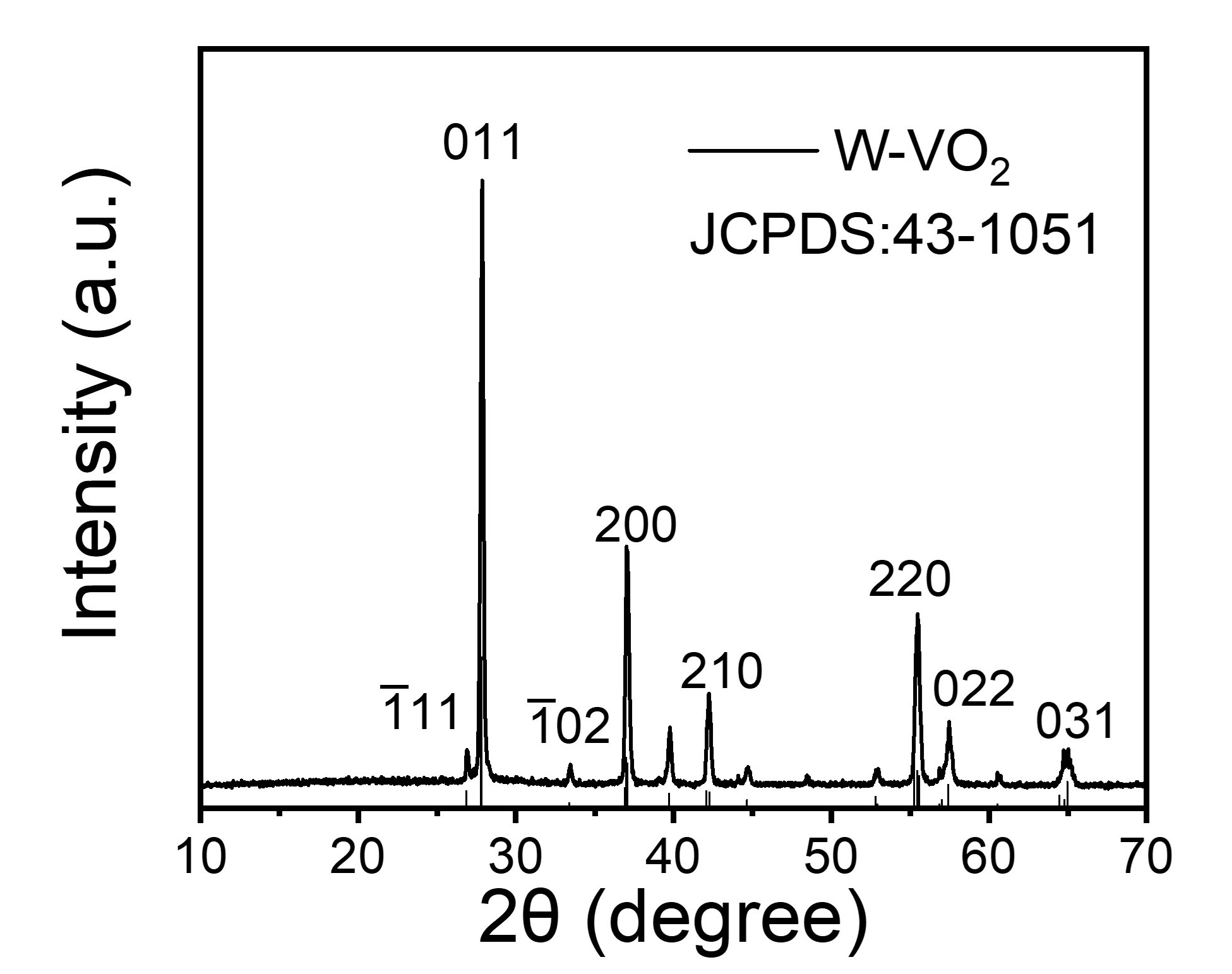


**Supplementary Figure 19. XRD patterns of W doped VO_2_ NWs.** Source data are provided as a Source Data file.

**Supplementary note 14. Fabrication and optical characterization of thermochromic films (TC and WRT films)**

**Supplementary Table 8.** The preparation conditions of thermochromic films (TC and WRT films).

|  | **TC1** | | **TC2** | **TC3** | **WRT1** | **WRT2** | **WRT3** | **WRT4** |
| --- | --- | --- | --- | --- | --- | --- | --- | --- |
| **W-VO_2_-1** | 1.0 mL | 0 mL | | 0 mL | 0.5 mL | 0.5 mL | 0 mL | 0.33 mL |
| **W-VO_2_-2** | 0 mL | 1.0 mL | | 0 mL | 0.5 mL | 0 mL | 0.5 mL | 0.33 mL |
| **W-VO_2_-3** | 0 mL | 0 mL | | 1.0 mL | 0 mL | 0.5 mL | 0.5 mL | 0.33 mL |


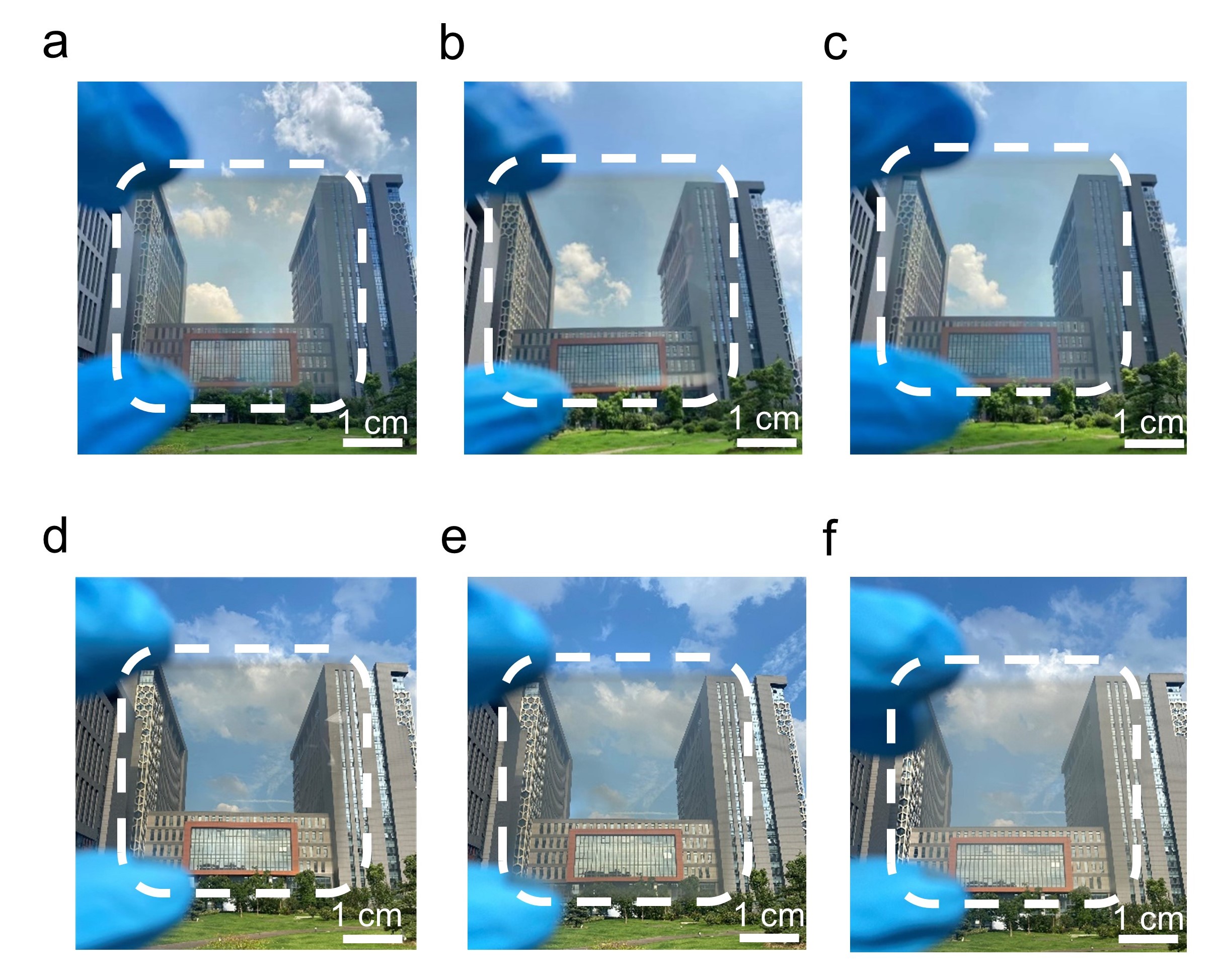


**Supplementary Figure 20. Photographs of thermochromic films with different types of W-VO_2_ NWs. a, b, c,** The thermochromic films TC1, TC2, and TC3 films. **d, e, f,** The thermochromic mixture films WRT1, WRT2, and WRT3 films. the areas covered with thermochromic films are denoted with the white imaginary line.


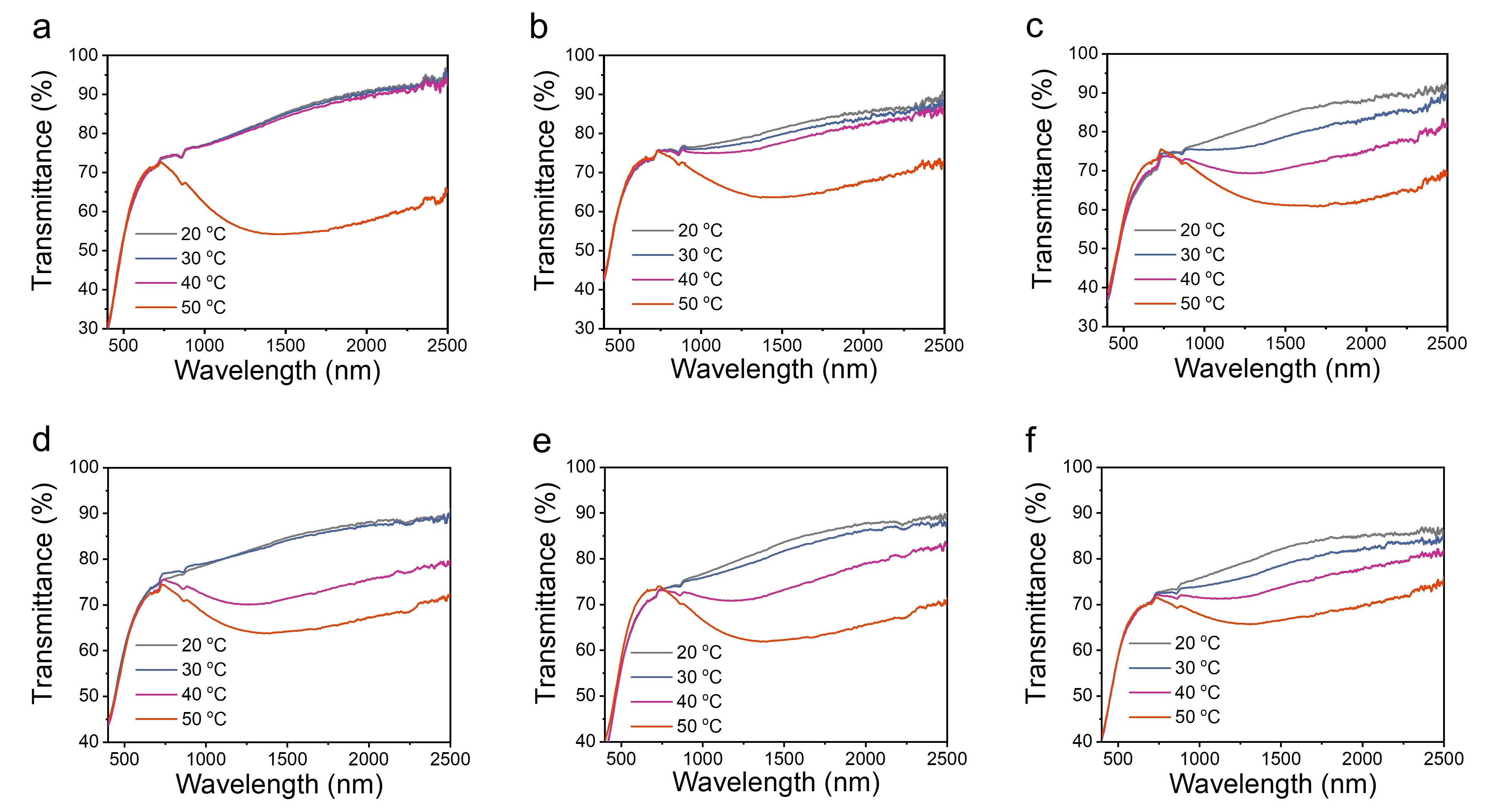


**Supplementary Figure 21. Thermochromic properties of thermochromic films with different types of W-VO_2_ NWs. a, b, c,** The transmittance spectra of TC1, TC2, and TC3 films. **d, e, f,** The transmittance spectra of WRT1, WRT2, and WRT3 films. Source data are provided as a Source Data file.

**Supplementary Table 9.** Integrated optical transmittance (T) of the TC amd WRT films in the VIS and NIR regions at different temperature.

| **VIS**  **NIR** | **TC1** | **TC2** | **TC3** | **WRT1** | **WRT2** | **WRT3** | **WRT4** |
| --- | --- | --- | --- | --- | --- | --- | --- |
| **20 ^o^C** | 60.0%/  85.7% | 66.1%/  82.2% | 61.2%/  84.3% | 65.7%/  84.5% | 62.0%/  83.5% | 62.9%/  81.7% | 65.9%/  87.5% |
| **30 ^o^C** | 60.1%/  85.4% | 66.2%/  80.6% | 61.8%/  80.3% | 66.1%/  84.2% | 62.0%/  82.1% | 62.9%/  79.0% | 66.2%/  86.4% |
| **40 ^o^C** | 60.2%/  84.8% | 66.3%/  79.3% | 61.8%/  71.5% | 65.3%/  73.8% | 62.1%/  75.7% | 62.9%/  75.3% | 66.3%/  78.0% |
| **50 ^o^C** | 60.2%/  65.8% | 66.5%/  67.6% | 63.7%/  64.4% | 65.1%/  67.1% | 64.2%/  65.6% | 63.0%/  68.9% | 66.6%/  62.6% |

**Supplementary Table 10.** Solar irradiance transmittance of the TC and WRT films in the VIS and NIR regions at different temperature.

| **VIS**  **NIR** | **TC1** | **TC2** | **TC3** | **WRT1** | **WRT2** | **WRT3** | **WRT4** |
| --- | --- | --- | --- | --- | --- | --- | --- |
| **20 ^o^C** | 59.7%/  78.5% | 65.9%/  77.8% | 60.9%/  78.5% | 65.7%/  80.0% | 61.7%/  77.9% | 62.8%/  76.8% | 65.6%/  82.2% |
| **30 ^o^C** | 59.9%/  78.5% | 66.0%/  76.9% | 61.5%/  76.0% | 65.4%/  79.7% | 61.8%/  76.9% | 62.8%/  74.8% | 65.9%/  81.7% |
| **40 ^o^C** | 59.9%/  78.2% | 66.0%/  75.8% | 61.5%/  71.7% | 64.7%/  72.5% | 61.8%/  72.3% | 62.7%/  72.1% | 66.0%/  76.0% |
| **50 ^o^C** | 60.0%/  62.0% | 66.3%/  69.0% | 63.4%/  67.8% | 64.7%/  68.1% | 64.0%/  66.8% | 62.8%/  68.1% | 66.3%/  67.7% |

**Supplementary note 15.** **Comparison of ordered and disordered structures in WRT films**


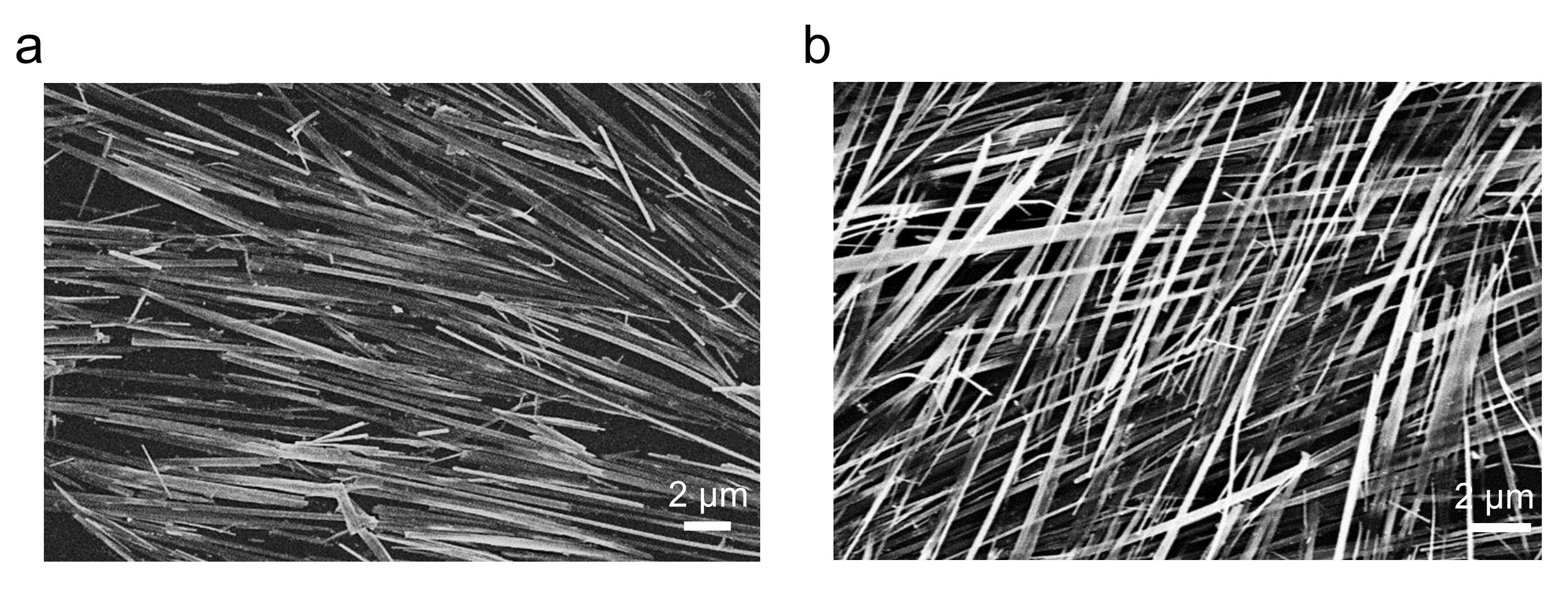


**Supplementary Figure 22.** **Ordered arrangement of W-VO_2_ NWs assemblies. a, b,** SEM images of WRT film with a monolayer of W doped VO_2_ NWs. **b,** SEM images of WRT film with two layers of W doped VO_2_ NWs network structure.


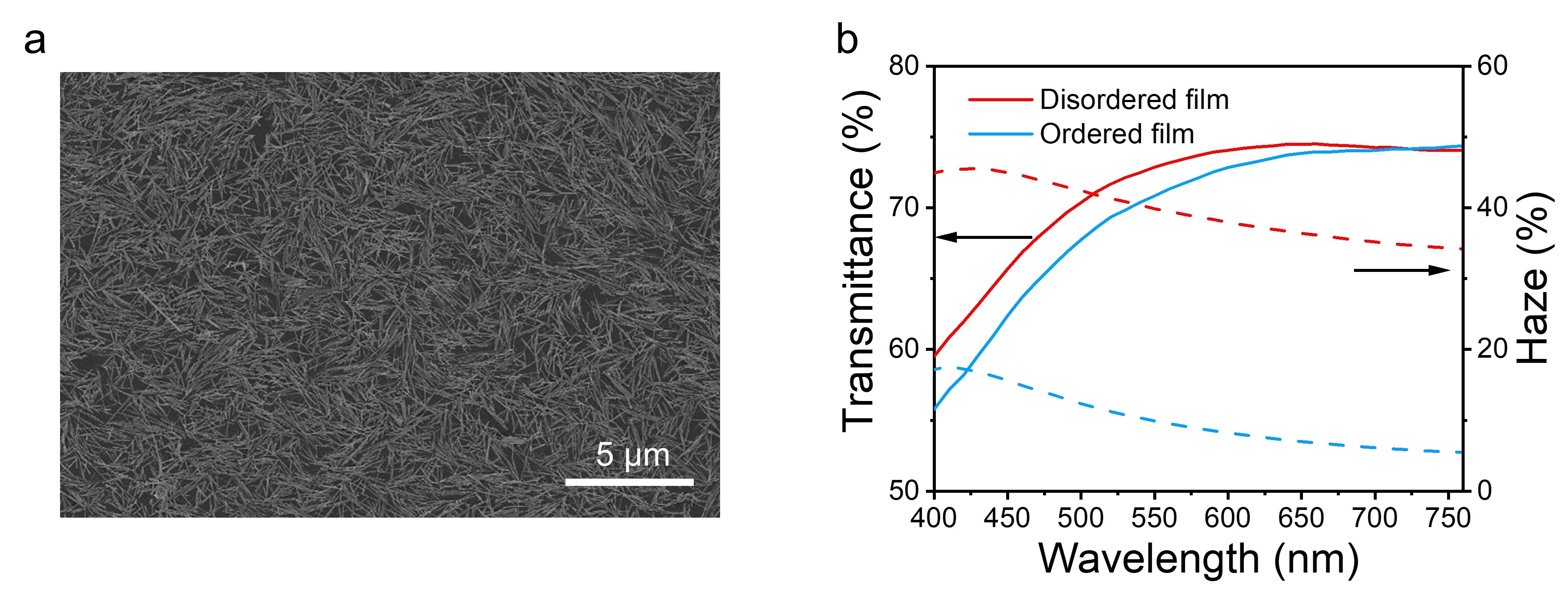


**Supplementary Figure 23. Comparison of ordered and disordered structures. a,** SEM image of disorder spraying W-VO_2_ mixture film. **b,** Optical transmittance and haze of the ordered and disordered structures in WRT films. Source data are provided as a Source Data file.

**Supplementary note 16. The film with two layers of W-doped VO_2_ NWs (WRT4-2L)**


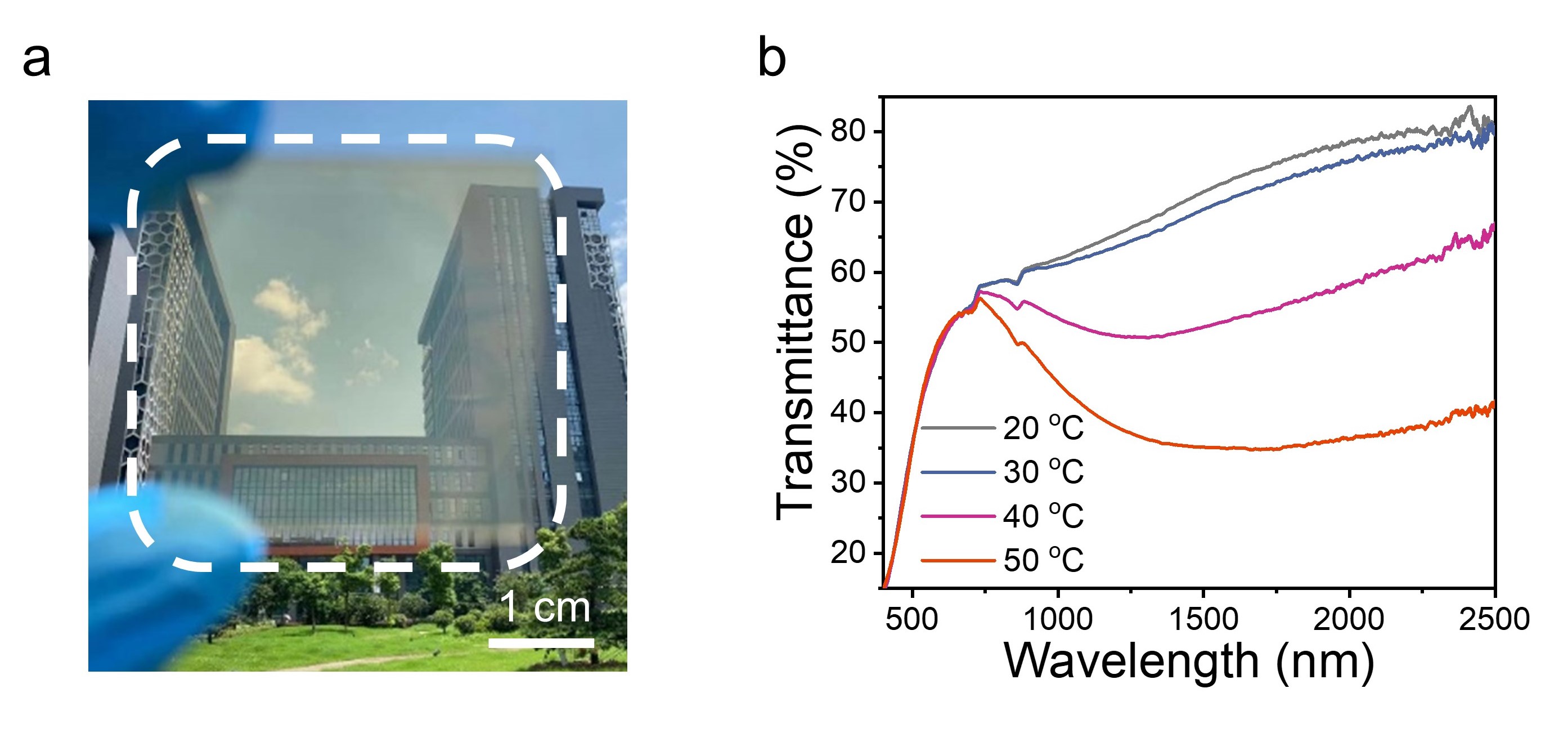


**Supplementary Figure 24.** **Characterization of films with two layers of W-doped VO_2_ NWs.** **a,** Photograph of film with two layers of W-doped VO_2_ NWs. **b,** The transmittance spectra of film with two layers of W-doped VO_2_ NWs. Source data are provided as a Source Data file.

**Supplementary note 17.** **The temperature of the indoor blackbody in the model chamber with a window installed with glass, TC, and TCM films under direct simulated sunlight**


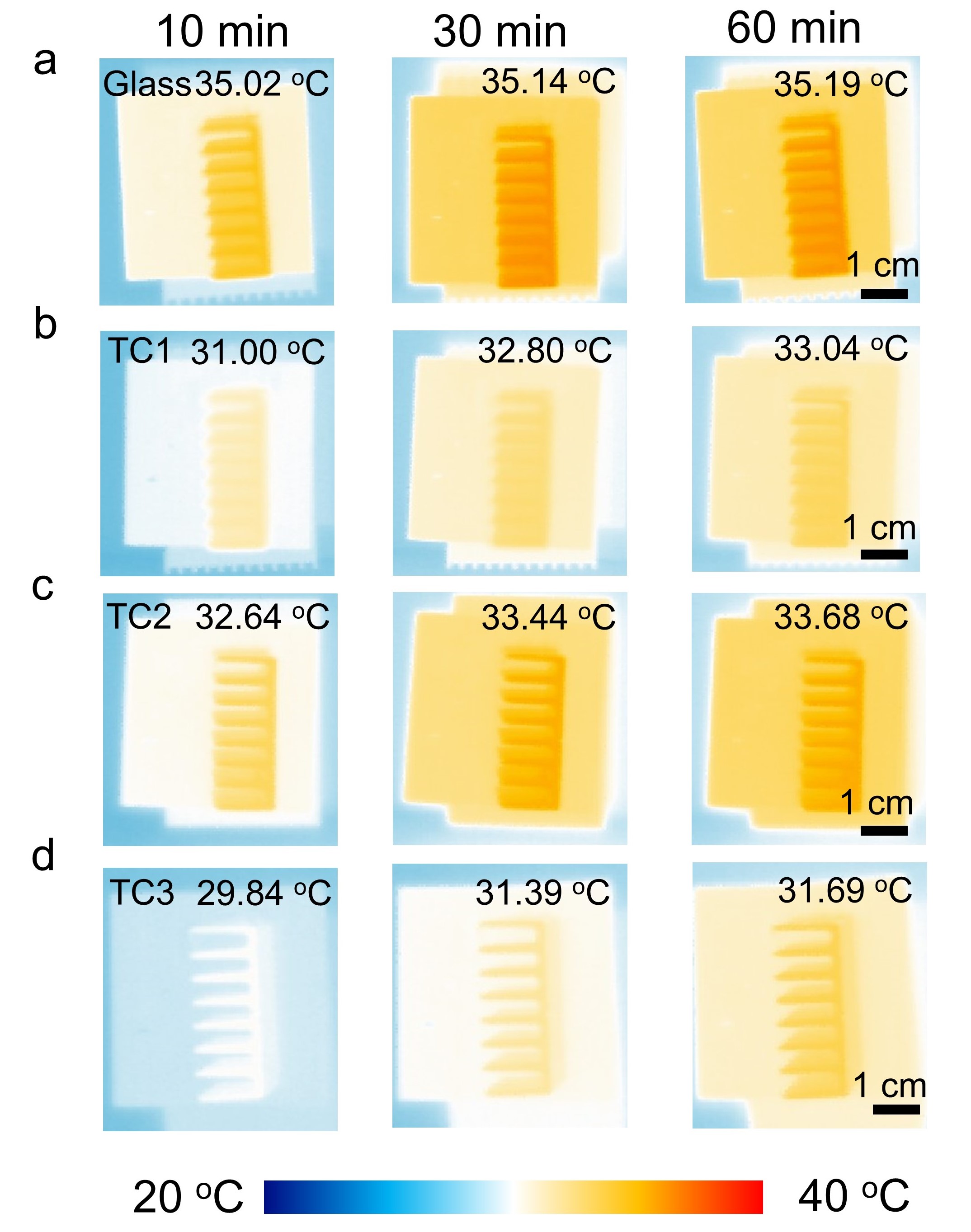


**Supplementary Figure 25. The cooling effect of normal glass and thermochromic windows. a-d,** The time-dependent infrared images of the indoor blackbody in the model chamber with a window installed with bare glass, TC1, TC2, and TC3 films, respectively.


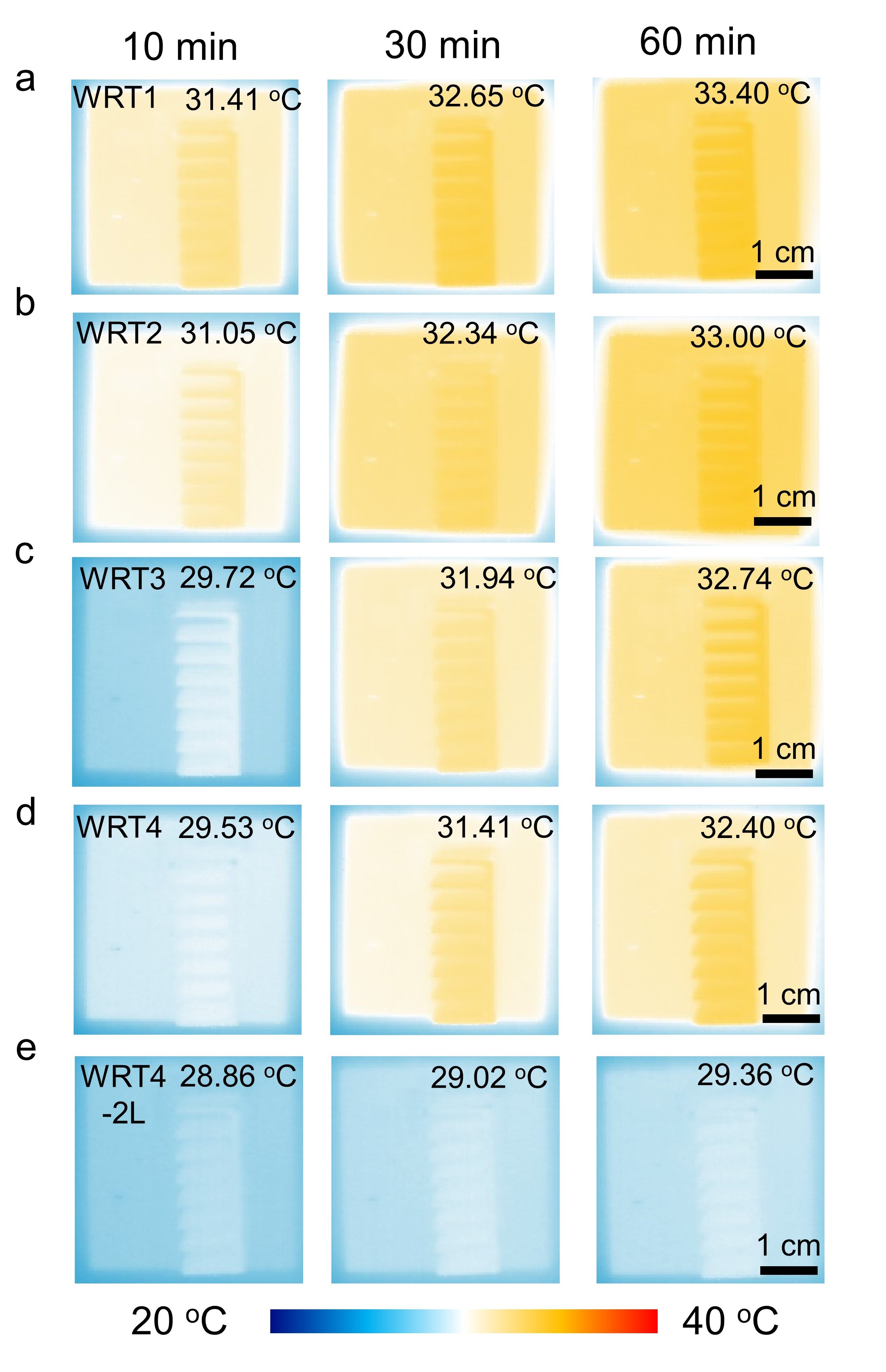


**Supplementary Figure 26. The cooling effect of wide response-range thermochromic windows. a-e,** The time-dependent infrared images of the indoor blackbody in the model chamber with a window installed with WRT1, WRT2, WRT3, WRT4, and WRT4-2L films, respectively.

**Supplementary note 18.** **The temperature of WRT4 films and indoor blackbody under different intensities of direct simulated sunlight**


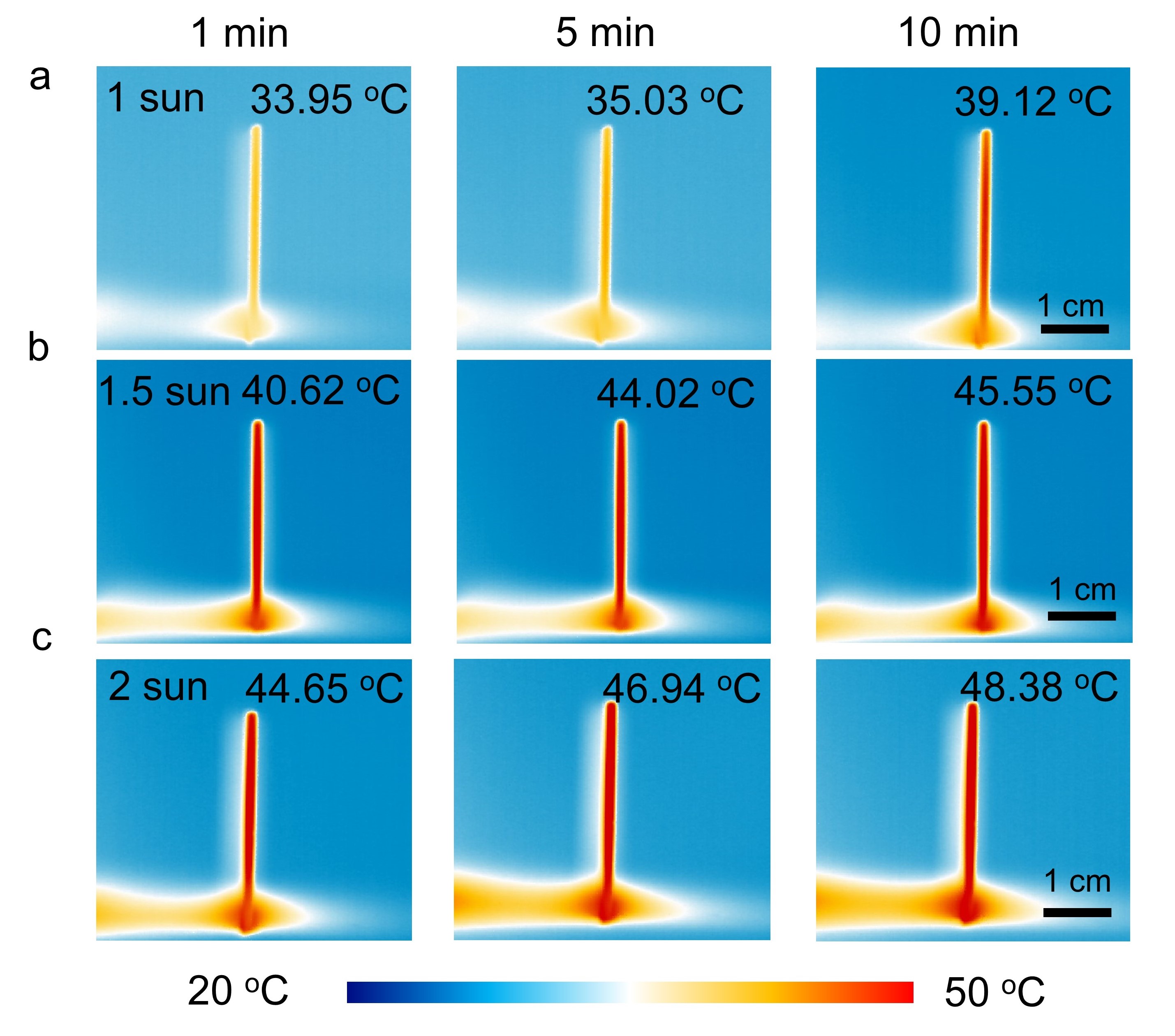


**Supplementary Figure 27. The temperature of wide response-range thermochromic windows under different light intensities. a,** 100 mW cm^-2^ direct simulated sunlight (1 sun). **b,** 150 mW cm^-2^ direct simulated sunlight (1.5 sun). **c,** 200 mW cm^-2^ direct simulated sunlight (2 sun).


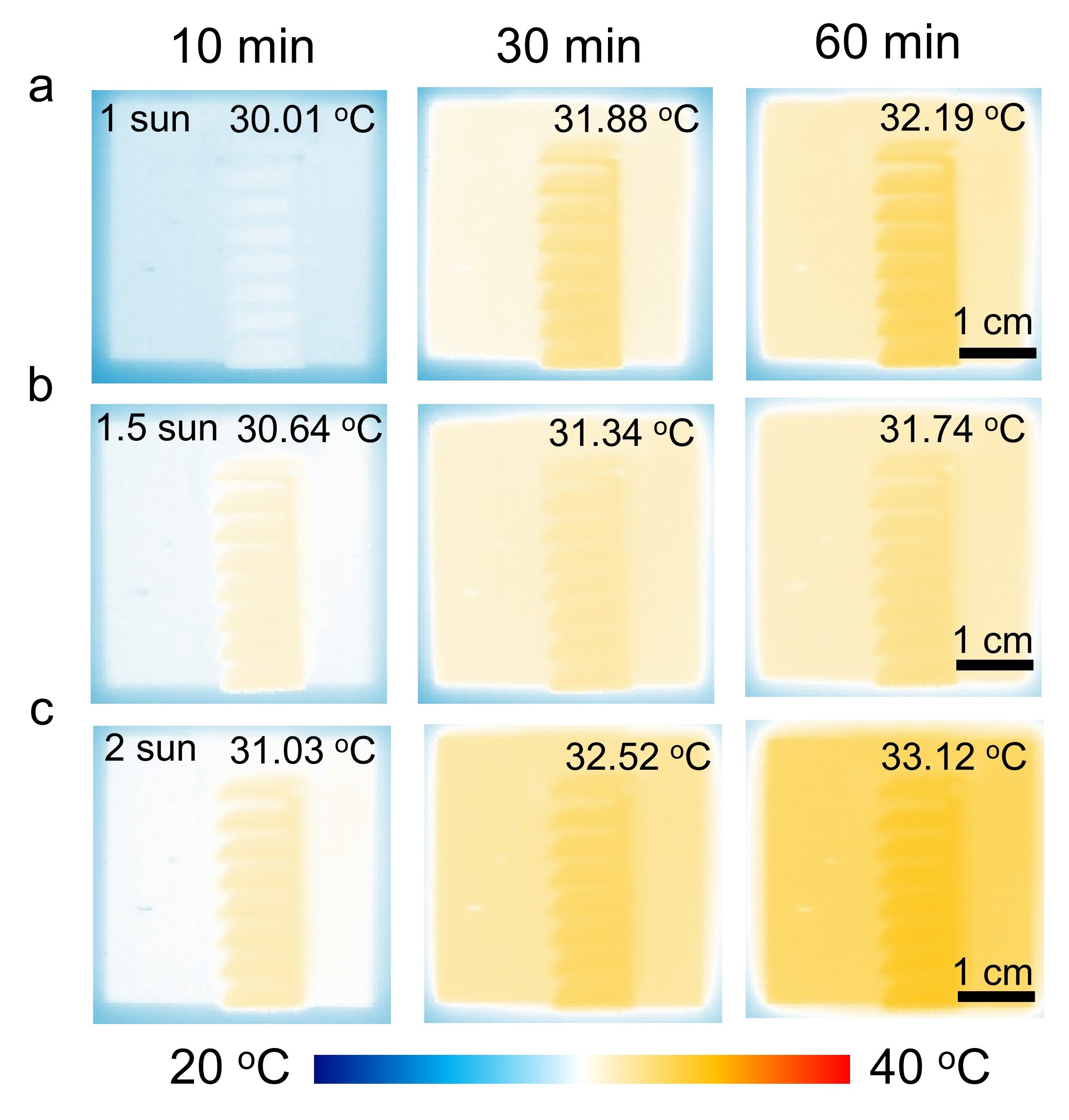


**Supplementary Figure 28. The cooling effect of wide response-range thermochromic windows under different light intensities. a,** 100 mW cm^-2^ direct simulated sunlight. **b,** 150 mW cm^-2^ direct simulated sunlight. **c,** 200 mW cm^-2^ direct simulated sunlight.


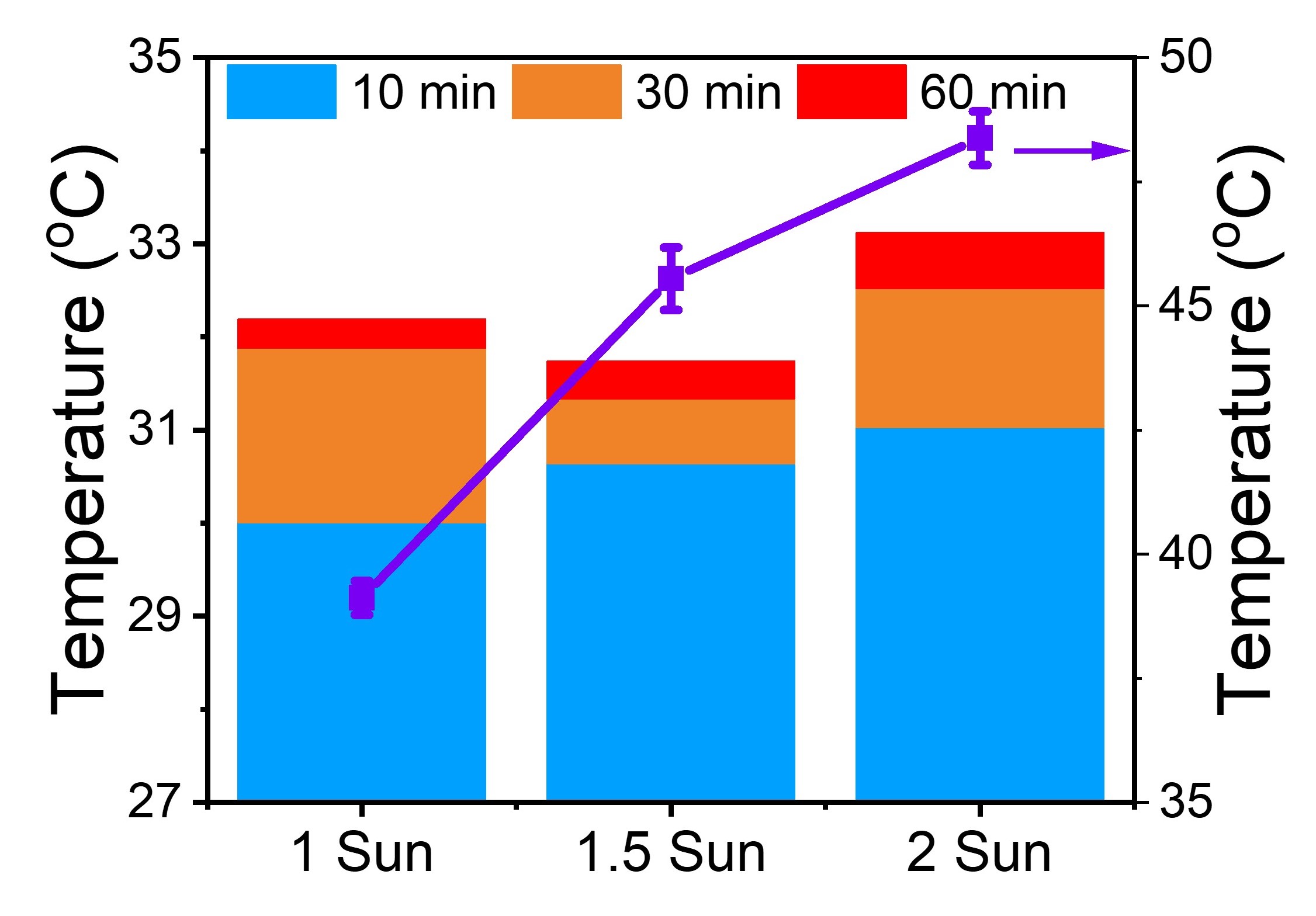


**Supplementary Figure 29.The Variation of temperature with time.** The temperature of blackbody in the model chamber installed with TCM4 film under the simulated illumination of different intensities. The corresponding error bar represents the standard deviation. For calculation, the cooling effect of each sample was measured three times. Source data are provided as a Source Data file.

**Supplementary note 19.** **Stability of the cooling performance of WRT4 film**


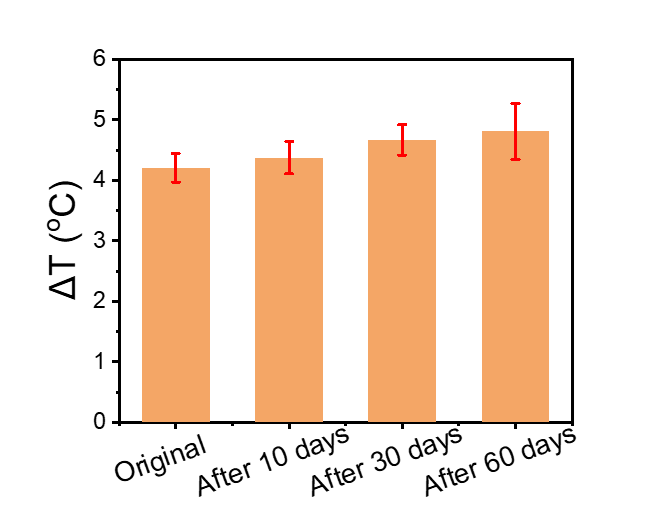


**Supplementary Figure 30.** **The stability of the cooling performance of WRT4 film.** The variation of cooling properties of WRT4 film exposed to air for the different time. The corresponding error bar represents the standard deviation. For calculation, the cooling effect of each sample was measured three times. Source data are provided as a Source Data file.

**Supplementary note 20. Fabrication of SLE4 and TCM4 films with a large area**

**Supplementary Table 11.** The fabrication costs of the materials in the unit area of SLE4 film.

|  | **Au**  **NRs** | | **Ag**  **NWs** | | **W_18_O_49_**  **NWs** | **DMF+**  **CHCl_3_** | **Substrate** | **labor cost** | **Total** |
| --- | --- | --- | --- | --- | --- | --- | --- | --- | --- |
| **Materials price** | 5.23 dollar m^-2^ | 0.079 dollar m^-2^ | | 22.08  dollar m^-2^ | | 0.25  dollar m^-2^ | 1.583  dollar m^-2^ | 5.96  dollar m^-2^ | 35.182  dollar m^-2^ |

**Supplementary Table 12.** The fabrication costs of the materials in the unit area of WRT4 film.

|  | **W-VO_2_-1 NWs** | **W-VO_2_-2 NWs** | **W-VO_2_-3 NWs** | **DMF+**  **CHCl_3_** | **Substrate** | **labor cost** | **Total** |
| --- | --- | --- | --- | --- | --- | --- | --- |
| **Materials price** | 0.098 dollar m^-2^ | 0.098 dollar m^-2^ | 0.098  dollar m^-2^ | 0.25  dollar m^-2^ | 1.589 dollar m^-2^ | 2.384  dollar m^-2^ | 4.511 dollar m^-2^ |


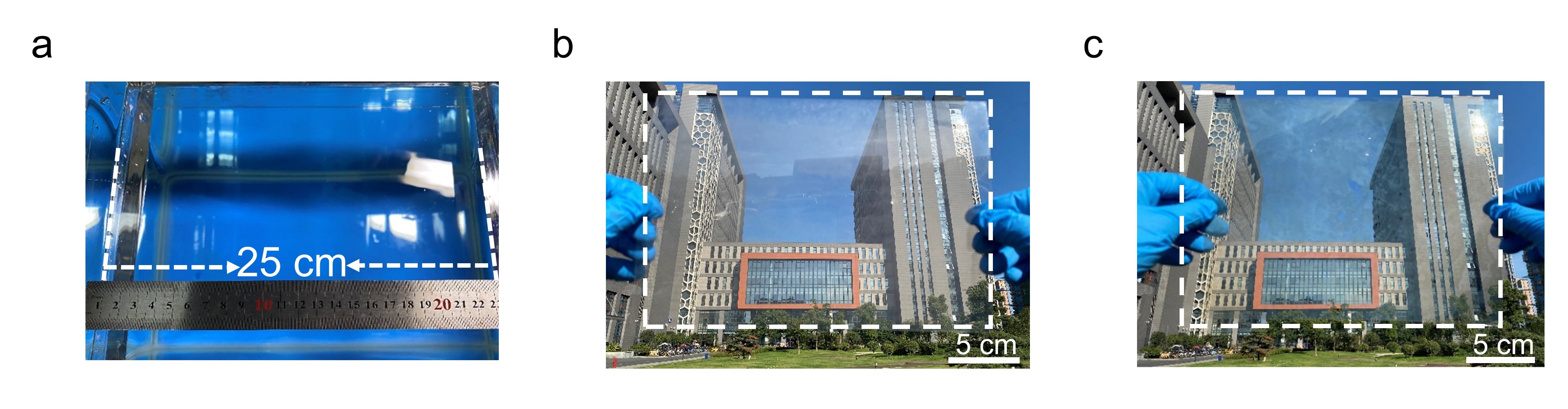


**Supplementary Figure 31. The smart windows with a large area. a,** Photograph of co-assembled nanowires monolayer on the water-air interface on a large-scale LB trough used for the fabrication of smart windows. **b, c,** Photographs of the large-area SLE and WRT smart windows with 25 × 20 cm^2^.

**Supplementary note 21. Simulation for energy-saving performance in buildings**

**
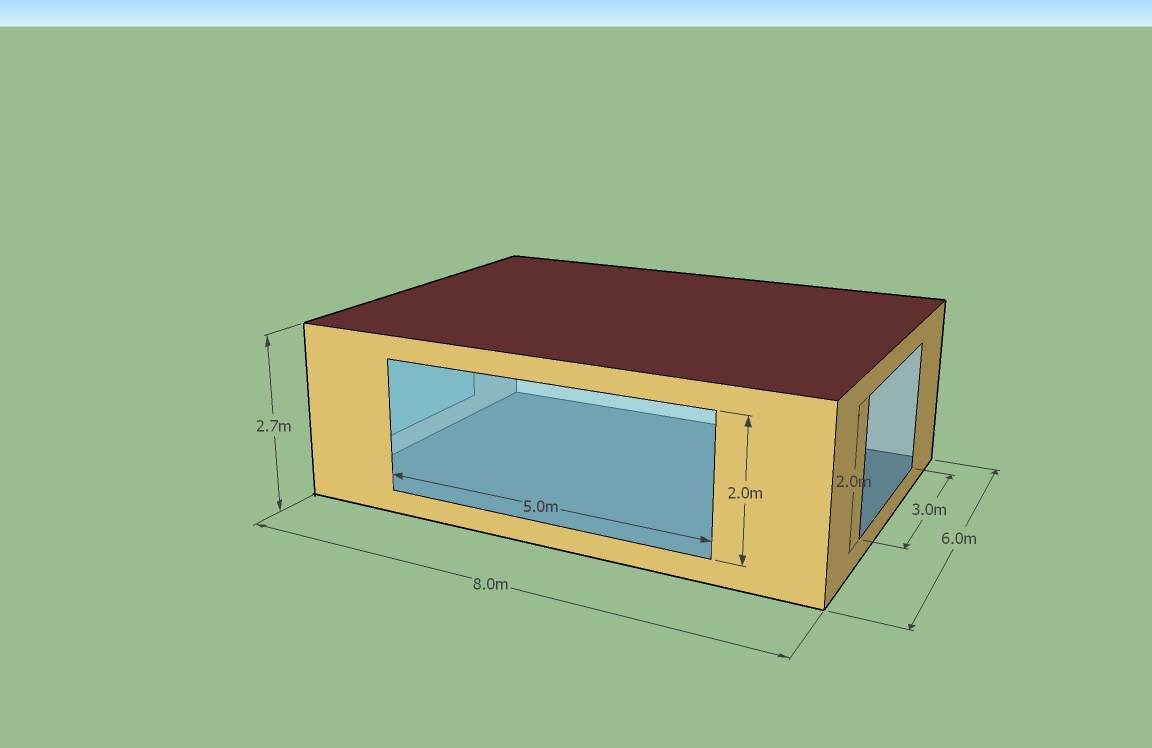
**

**Supplementary Figure 32.** **Schematic diagram of the building model used in the simulation.** An 8 × 6 × 2.7 m^3^ model house was built with two 5 × 2 m^2^ windows and two 3 × 2 m^2^ windows on four walls.


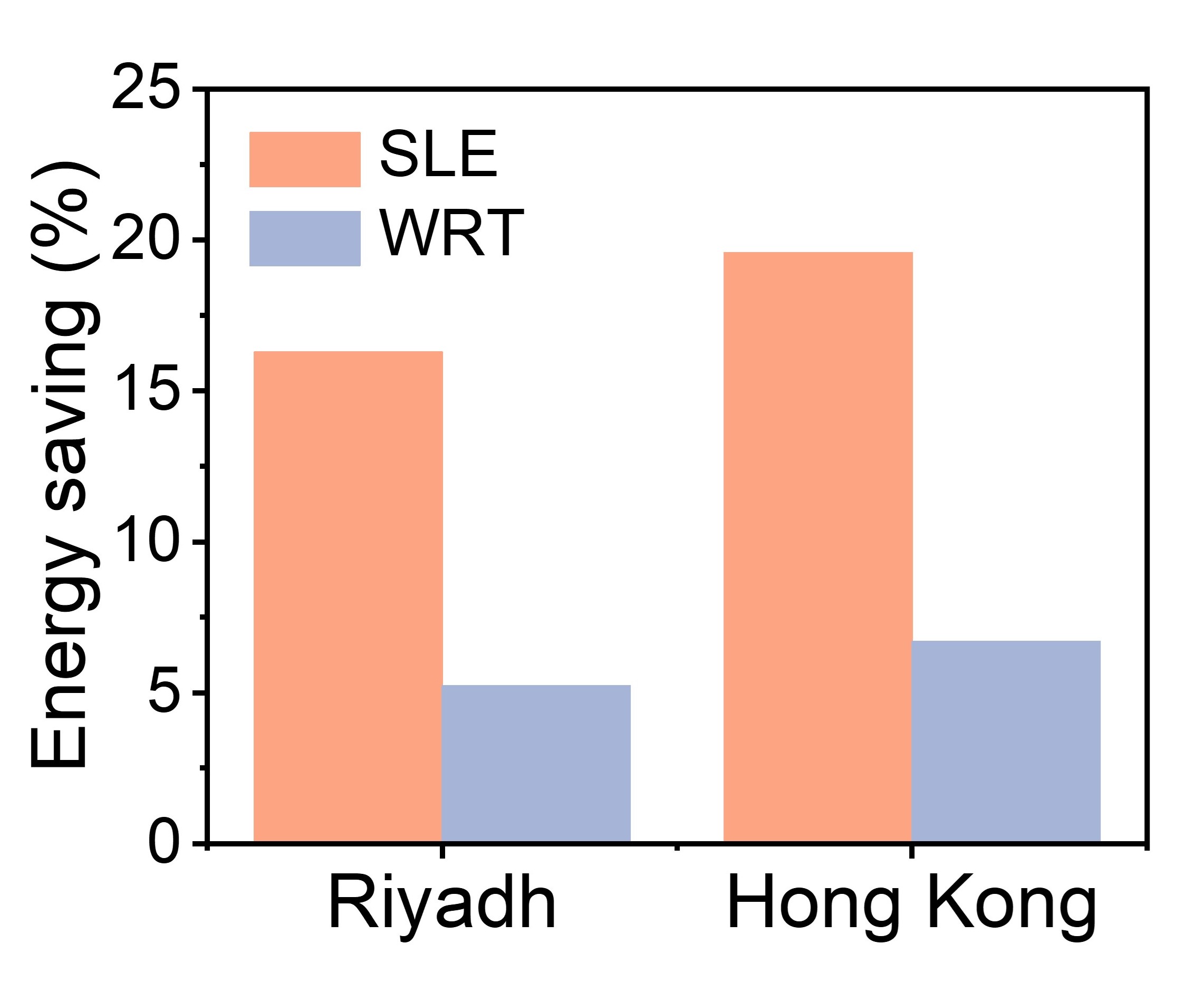


**Supplementary Figure 33.** **Energy saving of smart windows in different areas.** The total energy saving per year by using SLE and WRT smart windows over normal glass windows in Riyadh and Hong Kong, respectively. Source data are provided as a Source Data file.

**Supplementary note 22. Comparison of this work with different types of smart windows**


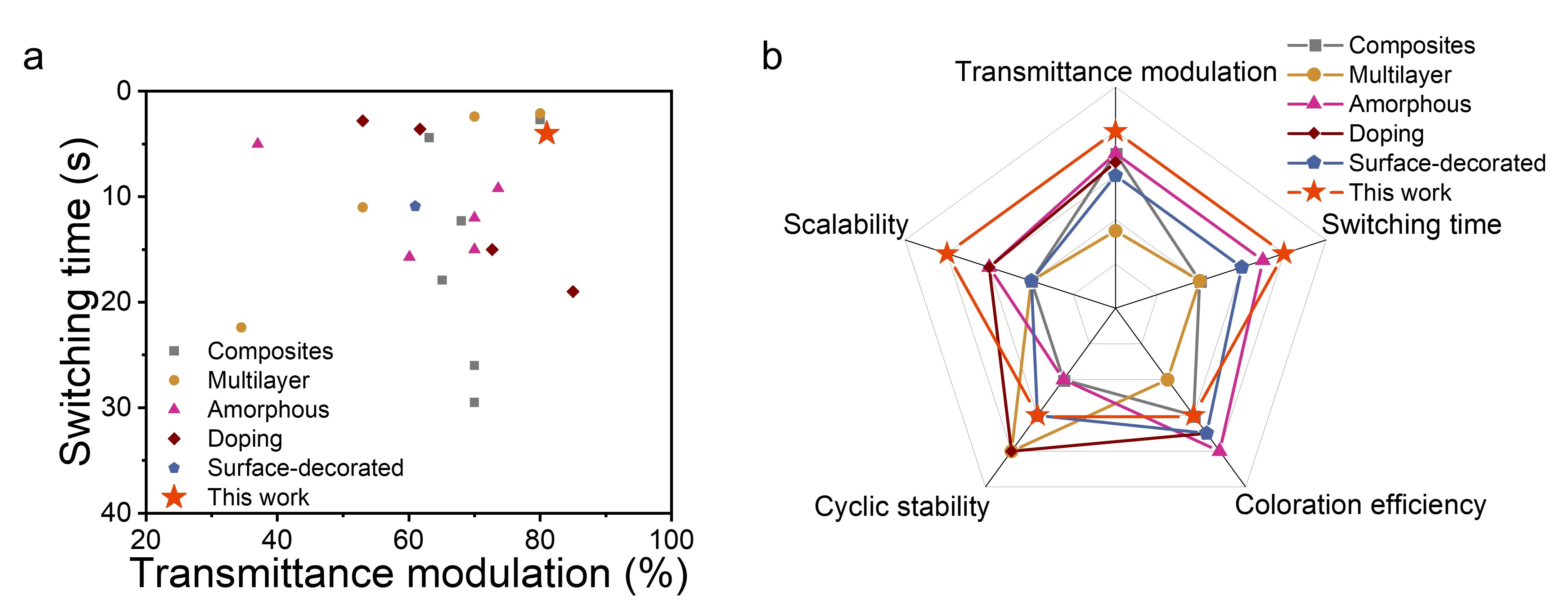


**Supplementary Figure 34.** **Comparison of this work with different types of electrochromic windows.** **a,** Summary of electrochromic performance (transmittance modulation and switching time) in some of the best reported and selected works. **b**, The radar plot in which the transmittance modulation, switching time, coloration efficiency, cyclic stability, and scalability of Tungsten Oxide based thermochromic windows are compared (Nanostructure: Ref 1, Multilayer: Ref 9, Amorphous: Ref 11, Doping: Ref 16, Surface-decorated: Ref 20). Source data are provided as a Source Data file.


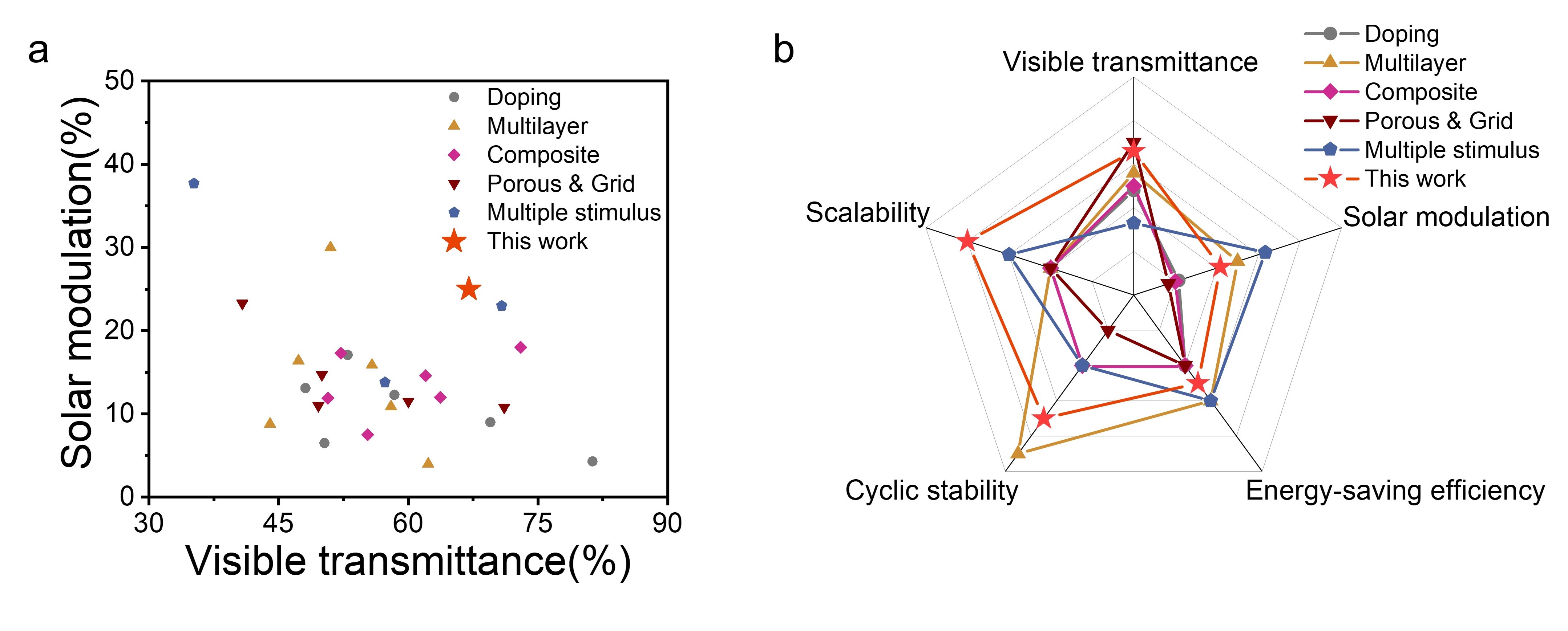


**Supplementary Figure 35. Comparison of this work with different types of thermochromic windows based on VO_2_.** **a,** Summary of thermochromic performance (visible transmittance and solar modulation) in some of the best reported and selected works. **b**, The radar plot in which the visible transmittance, solar modulation, energy-saving efficiency, cyclic stability, and scalability of VO_2_-based thermochromic windows are compared (Doping: Ref 24, Multilayer: Ref 30, Composite: Ref 38, Porous & Grid: Ref 41, Multiple stimulus: Ref 46). Source data are provided as a Source Data file.

**Supplementary references**

1. Gu, H. *et al.* Highly efficient, near-infrared and visible light modulated electrochromic devices based on polyoxometalates and W_18_O_49_ nanowires. *ACS Nano* **12**, 559-567 (2018).
2. Wang, J.-L., Lu, Y.-R., Li, H.-H., Liu, J.-W. & Yu, S.-H. Large area co-assembly of nanowires for flexible transparent smart windows. *J. Am. Chem. Soc.* **139**, 9921-9926 (2017).
3. Heo, S., Kim, J., Ong, G. K. & Milliron, D. J. Template-free mesoporous electrochromic films on flexible substrates from tungsten oxide nanorods. *Nano Lett.* **17**, 5756-5761 (2017).
4. Li, H., McRae, L. & Elezzabi, A. Y. Solution-processed interfacial PEDOT:PSS assembly into porous tungsten molybdenum oxide nanocomposite films for electrochromic applications. *ACS Appl. Mater. Interfaces* **10**, 10520-10527 (2018).
5. Nguyen, T. D. et al. Efficient near infrared modulation with high visible transparency using SnO_2_-WO_3_ nanostructure for advanced smart windows. *Adv. Optical Mater*. **7**, 1801389 (2019).
6. Qu, H. et al. Highly robust and flexible WO_3_·2H_2_O/ PEDOT films for improved electrochromic performance in near-infrared region. *Sol. Energy Mater Sol. Cells* **163**, 23-30 (2017).
7. Li, H., Lv, Y., Zhang, X., Wang, X. & Liu, X. High-performance ITO-free electrochromic films based on bi-functional stacked WO_3_/Ag/WO_3_ structures. *Sol. Energy Mater Sol. Cells* **136**, 86-91 (2015).
8. Xiao, L., Lv, Y., Dong, W., Zhang, N. & Liu, X. Dual-functional WO_3_ nanocolumns with broadband antireflective and high-performance flexible electrochromic properties. *ACS Appl. Mater. Interfaces* **8**, 27107-27114 (2016).
9. Najafi-Ashtiani, H., Akhavan, B., Jing, F. & Bilek, M. M. Transparent conductive dielectric-metal-dielectric structures for electrochromic applications fabricated by high-power impulse magnetron sputtering. *ACS Appl. Mater. Interfaces* **11**, 14871-14881 (2019).
10. Dong, W. et al. Bifunctional MoO3-WO_3_/Ag /MoO3-WO_3_ films for efficient ITO–free electrochromic devices. *ACS Appl. Mater. Interfaces* **8**, 33842-33847 (2016).
11. Cheng, W. et al. Photodeposited amorphous oxide films for electrochromic windows. *Chem* **4**, 821-832 (2018).
12. Huo, X. et al. Bifunctional aligned hexagonal/amorphous tungsten oxide core/shell nanorod arrays with enhanced electrochromic and pseudocapacitive performance. *J. Mater. Chem. A* **7**, 16867-16875 (2019).
13. Zhang, S. et al. Amorphous and porous tungsten oxide films for fast‐switching dual‐band electrochromic smart windows. *Adv. Optical Mater.* **11**, 2202115 (2023).
14. Li, Z. et al. Efficient electrochromic efficiency and stability of amorphous/crystalline tungsten oxide film. *J. Alloys Compd.* **930**, 167405 (2023).
15. Wang, J. et al. Amorphous mixed-vanadium-tungsten oxide films as optically passive ion storage materials for solid-state near-infrared electrochromic devices. *ACS Appl. Mater. Interfaces* **15**, 7120-7128 (2023).
16. Zhan, Y. et al. Ti-doped WO_3_ synthesized by a facile wet bath method for improved electrochromism. *J. Mater. Chem. C* **5**, 9995-10000 (2017).
17. Koo, B.-R., Kim, K.-H. & Ahn, H.-J. Switching electrochromic performance improvement enabled by highly developed mesopores and oxygen vacancy defects of Fe-doped WO_3_ films. *Appl. Surf. Sci.* **453**, 238-244 (2018).
18. Wang, W. et al. Niobium doped tungsten oxide mesoporous film with enhanced electrochromic and electrochemical energy storage properties. *J. Colloid. Interf. Sci* **535**, 300-307 (2019).
19. Zhou, J., Wei, Y., Luo, G., Zheng, J. & Xu, C. Electrochromic properties of vertically aligned Ni-doped WO_3_ nanostructure films and their application in complementary electrochromic devices. *J. Mater. Chem. C* **4**, 1613-1622 (2016).
20. Xu, J. et al. Electrochromic-tuned plasmonics for photothermal sterile window. *ACS Nano* **12**, 6895-6903 (2018).
21. Wang, N., Liu, S., Zeng, X., Magdassi, S. & Long, Y. Mg/W-codoped vanadium dioxide thin films with enhanced visible transmittance and low phase transition temperature. *J. Mater. Chem. C* **3**, 6771-6777 (2015).
22. Shen, N. et al. The synthesis and performance of Zr-doped and W–Zr-codoped VO_2_ nanoparticles and derived flexible foils. *J. Mater. Chem. A* **2**, 15087-15093 (2014).
23. Zhang, Z. et al. Thermochromic VO_2_ thin films: Solution-based processing, improved optical properties, and lowered phase transformation temperature. *Langmuir* **26**, 10738-10744 (2010).
24. Dai, L. et al. F-doped VO_2_ nanoparticles for thermochromic energy-saving foils with modified color and enhanced solar-heat shielding ability. *Phys. Chem. Chem. Phys.* **15**, 11723-11729 (2013).
25. Chen, S. et al. The visible transmittance and solar modulation ability of VO_2_ flexible foils simultaneously improved by Ti doping: An optimization and first principle study. *Phys. Chem. Chem. Phys.* **15**, 17537-17543 (2013).
26. Dietrich, M. K. et al. Influence of doping with alkaline earth metals on the optical properties of thermochromic VO_2_. *J. Appl. Phys.* **117**, 185301 (2015).
27. Zhang, Z. et al. Solution-based fabrication of vanadium dioxide on f: SnO_2_ substrates with largely enhanced thermochromism and low-emissivity for energy-saving applications. *Energy Environ. Sci.* **4**, 4290-4297 (2011).
28. Liu, C. et al. Index-tunable anti-reflection coatings: Maximizing solar modulation ability for vanadium dioxide-based smart thermochromic glazing. *J. Alloys Compd.* **731**, 1197-1207 (2018).
29. Chen, Z. et al. VO_2_-based double-layered films for smart windows: Optical design, all-solution preparation and improved properties. *Energy Mater. Sol. Cells* **95**, 2677-2684 (2011).
30. Chang, T. et al. Mitigating deterioration of vanadium dioxide thermochromic films by interfacial encapsulation. *Matter* **1**, 734-744 (2019).
31. Liu, C., Wang, N. & Long, Y. Multifunctional overcoats on vanadium dioxide thermochromic thin films with enhanced luminous transmission and solar modulation, hydrophobicity and anti-oxidation. *Appl. Surf. Sci.* **283**, 222-226 (2013).
32. Hao, Q. et al. VO_2_/TiN plasmonic thermochromic smart coatings for room-temperature applications. *Adv. Mater.* **30**, 1705421 (2018).
33. Gao, Y. et al. Enhanced chemical stability of VO_2_ nanoparticles by the formation of SiO_2_/ VO_2_ core/shell structures and the application to transparent and flexible VO_2_-based composite foils with excellent thermochromic properties for solar heat control. *Energy Environ. Sci.* **5**, 6104-6110 (2012).
34. Zhu, J. et al. Vanadium dioxide nanoparticle-based thermochromic smart coating: High luminous transmittance, excellent solar regulation efficiency, and near room temperature phase transition. *ACS Appl. Mater. Interfaces* **7**, 27796-27803 (2015).
35. Liu, C. et al. VO_2_/Si–Al gel nanocomposite thermochromic smart foils: Largely enhanced luminous transmittance and solar modulation. *J. Colloid Interface Sci.* **427**, 49-53 (2014).
36. Chen, Z., Cao, C., Chen, S., Luo, H. & Gao, Y. Crystallised mesoporous TiO_2_ (a)-VO_2_ (M/R) nanocomposite films with self-cleaning and excellent thermochromic properties. *J. Mater. Chem. A* **2**, 11874-11884 (2014).
37. Zhu, J. et al. Hybrid films of VO_2_ nanoparticles and a nickel (ii)-based ligand exchange thermochromic system: Excellent optical performance with a temperature responsive colour change. *New J. Chem*. **41**, 830-835 (2017).
38. Moot, T., Palin, C., Mitran, S., Cahoon, J. F. & Lopez, R. Designing plasmon‐enhanced thermochromic films using a vanadium dioxide nanoparticle elastomeric composite. *Adv. Optical Mater.* **4**, 578-583 (2016).
39. Ke, Y. et al. Two-dimensional SiO_2_/ VO_2_ photonic crystals with statically visible and dynamically infrared modulated for smart window deployment. *ACS Appl. Mater. Interfaces* **8**, 33112-33120 (2016).
40. Cao, X. et al. Nanoporous thermochromic VO_2_(M) thin films: Controlled porosity, largely enhanced luminous transmittance and solar modulating ability. *Langmuir* **30**, 1710-1715 (2014).
41. Zhuang, B. et al. 3D ordered macroporous VO_2_ thin films with an efficient thermochromic modulation capability for advanced smart windows. *Adv. Optical Mater.* **7**, 1900600 (2019).
42. Zhou, C. et al. 3D printed smart windows for adaptive solar modulations. *Adv. Optical Mater.* **8**, 2000013 (2020).
43. Ke, Y. et al. Cephalopod-inspired versatile design based on plasmonic VO_2_ nanoparticle for energy-efficient mechano-thermochromic windows. *Nano Energy* **73**, 104785 (2020).
44. Shen, N. et al. Joule heating driven infrared switching in flexible VO_2_ nanoparticle films with reduced energy consumption for smart windows. *J. Mater. Chem. A* **7**, 4516-4524 (2019).
45. Chen, S. et al. Gate-controlled VO_2_ phase transition for high-performance smart windows. *Sci. Adv*. **5**, eaav6815 (2019).
46. Ke, Y. et al. Adaptive thermochromic windows from active plasmonic elastomers. *Joule* **3**, 858-871 (2019).
